# Long-term effects of catastrophic wind on southern US coastal forests: Lessons from a major hurricane

**DOI:** 10.1371/journal.pone.0243362

**Published:** 2021-01-06

**Authors:** Ajay Sharma, Santosh K. Ojha, Luben D. Dimov, Jason G. Vogel, Jarek Nowak

**Affiliations:** 1 West Florida Research and Education Center, University of Florida, Milton, Florida, United States of America; 2 Department of Biological and Environmental Sciences, Alabama A&M University, Normal, Alabama, United States of America; 3 Rubenstein School of Environment and Natural Resources, University of Vermont, Burlington, Vermont, United States of America; 4 School of Forest Resources and Conservation, University of Florida, Gainesville, Florida, United States of America; 5 Florida Forest Service, Florida Department of Agriculture and Consumer Services, Tallahassee, Florida, United States of America; Chinese Academy of Forestry, CHINA

## Abstract

Threats posed by windstorms are an increasing concern to forest managers in the southern United States (US). Studies suggest that the southern US will experience an increase in the occurrence as well as the intensity of windstorms, such as hurricanes, in the future. However, forest managers may have difficulty preparing for this future because there is limited understanding of how windstorms affect the structure and composition of forests over the long term. In this study, we evaluated the impacts of Hurricane Ivan, which made landfall in September 2004 near Gulf Shore, Alabama, impacting forests in the western Florida Panhandle and southwestern Alabama. We acquired the United States Department of Agriculture Forest Inventory and Analysis (FIA) plot data available for the period from 2002 to 2018 for the Ivan-affected area and classified the plots into 4 categories: (1). ND (No Disturbance), (2). NDBH (No Disturbance but Harvested), (3). ID (Disturbance caused by Hurricane Ivan), and (4). IDAH (Disturbance caused by Hurricane Ivan and Harvested). The plots that were damaged by Hurricane Ivan (ID and IDAH plots) had significantly (α = 0.05) (1) higher basal area, (2) higher quadratic mean diameter and height, (3) more diverse tree species composition (species richness and Shannon diversity index), (4) denser stocking of seedling and saplings, (5) lower proportion of dead trees or saplings, and (6) higher live aboveground biomass than the plots that were not damaged by the hurricane (ND and NDBH plots). Diverse stands were not necessarily more windstorm resistant. Species diversity in the overstory may not improve forest resistance to hurricane damage but may improve its resilience following the hurricane. The study suggests that managing stand structure through density management and stand improvement could be critical to windstorm resilience and resistance in the southern US forests.

## Introduction

Forests of the southern US are among the most ecologically and economically important forests in the world. The health and management of these forests is closely related to the economic wellbeing of not only the regional, but also the national and global economies. Being among the most productive forests in the world, southern US forests yield about 18% of the world’s pulpwood and 7% of its industrial wood, while comprising just 2% of the world’s forest area [1]. The southern US states are known as the nation’s “wood basket,” accounting for 63% of the total timber volume harvested in the US in 2011 [1, 2]. Forestry and the forest products industry generated $251.1 billion, or over 2.7% of the regional economic output, and supported 2% of all jobs in the southern US in 2012 [1]. The southern US region also includes the North American Coastal Plain (NACP), a global biodiversity hotspot [3]. In addition, these forests protect water quality, prevent erosion, help regulate climate, and provide opportunities for millions of people to hike, hunt, and experience natural beauty.

The sustainability of these forest services, however, is increasingly uncertain and threatened by changing climate and wind patterns. For example, several windstorms (hurricanes, tornadoes, etc.) have caused extensive damage and changes to these forests in recent decades [4–7]. Although windstorms, along with other natural disturbances, have historically shaped the structure and function of these ecosystems, the changing disturbance regimes, primarily driven by climate change, pose an unprecedented threat to these forests. Since the beginning of the Holocene to mid-20^th^ century, more than 40,000 tropical storms have been known to strike the northern Gulf coast of southern US [8]. Since 1995, however, the frequency and intensity of these windstorms in the Atlantic region have increased [9]. Further, climate model projections suggest that this trend will continue in the future because of global warming [10, 11]. In fact, 2020 is the most active Atlantic hurricane season on record in terms of number of tropical or subtropical storms [12]. This increasing frequency and intensity of hurricanes is likely outside of the southern forest ecosystem’s historic norms [13–15], which raises concerns about the sustainability and future of these forests.

Hurricanes affect forest ecosystems in a variety of ways. They cause widespread damage leading to the loss of timber and wildlife. For example, Hurricane Ivan in 2004, severely damaged north-west Florida and southwestern Alabama, causing a loss of more than $610 million (in 2004 dollars) worth of timber on approximately 1.1 million hectares in the state of Alabama alone [16]. More recently, in 2017 and 2018, vast areas of forest have experienced wind disturbance in the southern U.S. states of Florida and Georgia by hurricanes Irma and Michael, respectively. Hurricanes also lead to saltwater intrusion and storm surge flooding in coastal ecosystems [17], magnitude of which could be much higher in the future with increasing sea level rise caused by global warming [18]. Saltwater intrusion can cause mortality of trees, alter forest composition and lead to expansion of marshes into the coastal forested areas [17, 19, 20].

Ecologically, hurricane effects on forests can be complex, both adverse and beneficial, depending on the tree species and variable of interest. Reductions of forest canopy cover causes dramatic changes in site factors, leading to increased light availability and temperature, lower humidity, and enhanced exposure. Heavy rainfall during hurricanes helps alleviate the impacts of droughts and may provide young and/or stressed forests with a microclimate conducive to survival and growth. Fruits, flowers, and leaves may be damaged or removed for varying periods of time following hurricanes. Hurricane-caused disturbances may also prevent transition of mature forests to a late-successional stage, and sometimes enhance ecosystem productivity and structural diversity [8]. Additionally, stands that experience hurricane disturbance are vulnerable to invasive species [21, 22]. In effect, forest structure and composition shift as a result of hurricane disturbances. Assessing these forest structural and compositional changes following hurricanes over time is the key to understanding the complex ecological effects of hurricanes on forests [23]. This understanding can inform forest management approaches aimed at increasing forest resistance and resilience.

Several factors influence forest vulnerability and response to hurricane damage, with wind speed and forest structure being among the most important [24, 25]. Local topography and exposure of an area may cause little to significant effect on the windthrow damage to the forest stands [25, 26]. Mortality, in general, is more common in the mature age classes than in the juvenile and younger age classes [7, 27, 28]. Species may exhibit varying resistance and resilience to windstorms due to the differences in their stem, crown and root architecture, as well as stem strength and elasticity and leaf shape and texture [29]. In southern pine forests, for example, longleaf pine (*Pinus palustris*) has been found to be less affected than other species following hurricanes [30–34]. Stands of taller and older trees could also be more vulnerable to windthrow than stands of younger, shorter trees [25]. Stand density has varying effects on windstorm resilience depending on previous silvicultural treatments as well as the stage of stand development [24, 35, 36]. Rainfall amount and prior ground saturation may also play a role in the amount of windthrow as softer ground is conducive to tipping trees over. Saltwater intrusion and storm surge flooding caused by hurricanes could be influential factors affecting forest stands in coastal areas [17]. Over a large heterogenous area consisting of multiple forest types at different developmental stages, however, forest response following windstorms would be shaped by an interaction of all these factors. The current understanding of these complex spatial and temporal impacts of hurricanes on the structure and composition of forest ecosystems remains limited. For example, it is unclear how stand or species characteristics affect tree susceptibility to damage by hurricanes, and which structural and compositional forest attributes make them more resistant or resilient to the disturbance. Moreover, salvage harvest is common after hurricanes but it is unclear how this impacts succession. Answers to all these questions will help land managers to create and maintain forest conditions that will keep the stands more adaptive to the increasing uncertainty and changing disturbance regimes.

Several studies have made assessments of forest damage and responses after major storm events across the world (e.g., [25, 37–41]), with some specifically focused on the southern US (e.g., [7, 30, 31, 42–44]. Most of these studies have assessed damage soon after hurricanes and/or analyzed short-term changes to forest structure and function. Fewer studies, however, have reported on Hurricane Ivan or made comprehensive assessment of forest damage and responses over the long term. In this study, we utilized the United States Department of Agriculture (USDA) Forest Service’s Forest Inventory and Analysis (FIA) data [45] available for the Hurricane Ivan-affected area and comprehensively analyzed a wide suite of forest responses to the hurricane over a period of 14 years. The broad objectives of the study included: (1) identify the structural characteristics that make forest stands susceptible or resistant to hurricane disturbance, and (2) quantify the long-term change in the structure and composition of southern forests following Hurricane Ivan. The long-term FIA data provides objective and scientifically credible information on forest inventory and key ecosystem processes that allowed us to assess the status and trends of the study area forests pre- and post-hurricane on a periodic basis.

## Material and methods

### Hurricane Ivan and study area

**Fig 1:**
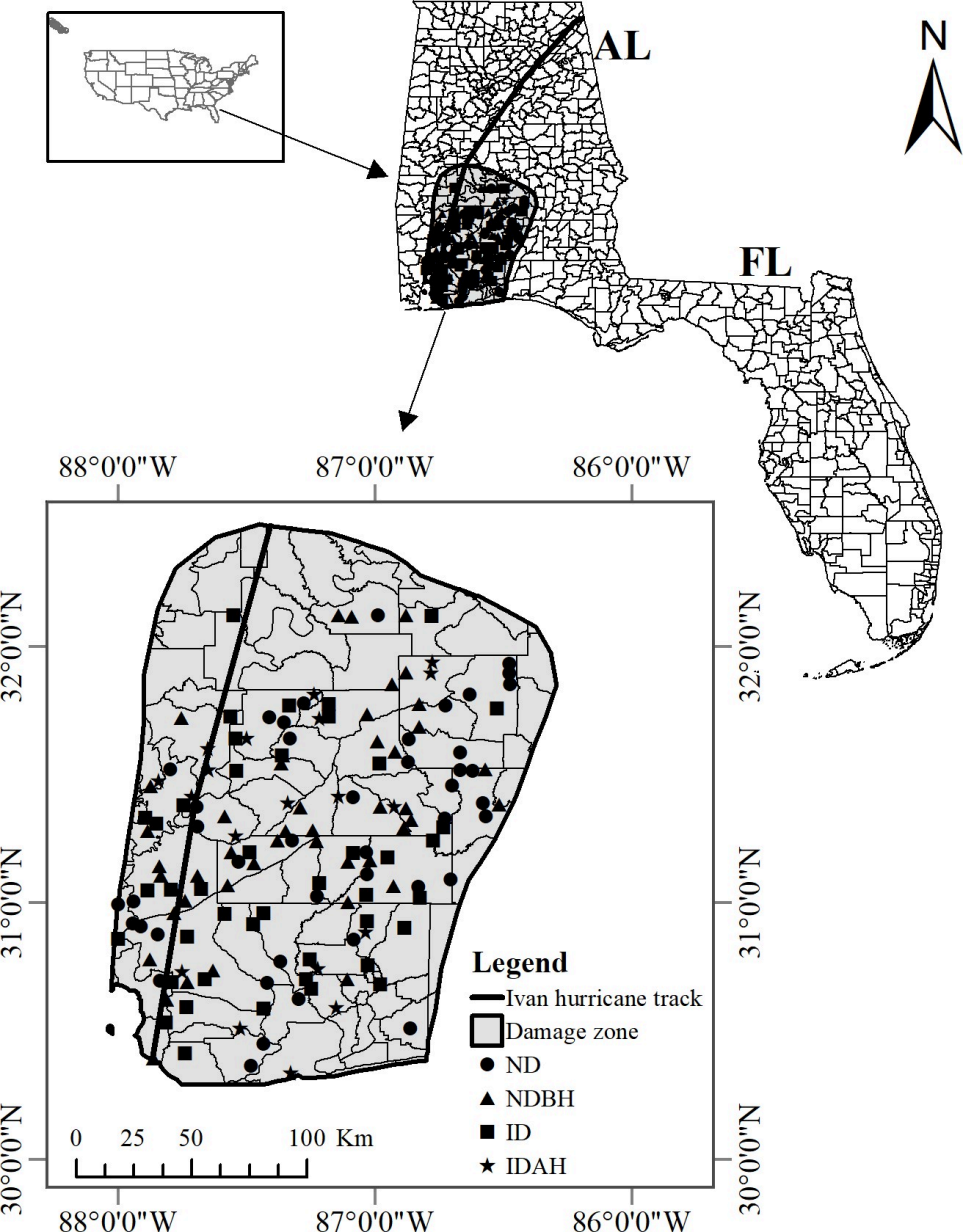
The study area included the area impacted by Hurricane Ivan in Alabama (AL) and Florida (FL). Sources: 1. Cartographic boundary shapefiles were obtained from the US Census Bureau (https://www.census.gov/geographies/mapping-files/time-series/geo/cartographic-boundary.html; last accessed 11.12.2020), and 2. Hurricane damage boundary map was adapted from the Alabama Forestry Commission [47].

Hurricane Ivan was a Cape Verde-type hurricane that originated from a tropical wave moving off the west coast of Africa on August 31, 2004. After a long track for more than 9,010 km over 22 days, 10 of which were spent as a major hurricane, on the morning of 16 September at around 2:10 am CDT, the hurricane made landfall near the Alabama-Florida border as a Category 3 hurricane with sustained winds of 193 km/h and a 4.3 m storm surge, causing tremendous wind and water damage in the area [46]. Timber losses were most significant in southwestern Alabama and west Florida Panhandle [16], which formed our study area (Fig 1). This area represented twelve southwest Alabama counties (Baldwin, Butler, Clarke, Coffee, Conecuh, Covington, Crenshaw, Escambia, Geneva, Mobile, Monroe, and Wilcox) and three western Florida Panhandle counties (Escambia, Santa Rosa, and Okaloosa) directly impacted by the storm. The primary FIA forest type groups of the study area are loblolly-shortleaf pine (*P*. *taeda-P*. *echinata)* (~40%), oak-hickory (*Quercus-Carya*), (~29%), oak-pine (*Quercus-Pinus*) (~12%), oak-gum-cypress (*Quercus-Nyssa-Taxodium*) (~9%), longleaf-slash pine (*P*. *palustris-P*. *elliottii*) (~6%), elm-ash-cottonwood (*Ulmus-Fraxinus-Populus*) (~3%), that constitute more than 99% of the forests of the area.

### Data source and description

Data on the storm track and the impacted forest area were obtained from the Alabama Forestry Commission [16, 47]. For this area, we extracted forestry data from the FIA database (FIADB version 7.0, https://www.fia.fs.fed.us). The contemporary FIA dataset is a national inventory program implemented by the USDA Forest Service (O’Connell et al., 2016), that uses a standard fixed-radius plot design [48] and common data collection procedures nationwide to assess forest inventory conditions on annual bases [49]. Under the FIA design, all forest and other land uses are sampled with one permanent plot established for every 2,428 ha of land [48]. A typical FIA plot consists of four 7.3 m fixed radius subplots, with three subplots spaced 36.6 m apart in a triangular arrangement and a subplot in the center [45]. Tree stems with a diameter at breast height (dbh) 1.37 m above the ground of ≥12.7 cm are measured in all subplots. Each subplot includes a 2.07 -m radius microplot for measuring saplings (2.5 cm ≤ dbh < 12.7 cm), seedlings (dbh < 2.5 cm), and other vegetation. In total, data are collected on more than 300 variables related to tree, stand, and site characteristics, as well as ownership. These data are used to report annual and periodic changes in the forests. For seedlings, only count or abundance data are available. In addition, the FIA records up to three disturbances that occurred on the plots since the previous measurement, or for new plots, within the previous 5 years. A disturbance, according to the FIA definition, is any event that caused damage or mortality to at least 25 percent of the trees, when the area affected by the disturbance is at least 0.4 hectares [45].

FIA database is the most comprehensive primary source of forest inventory data in the US and has been utilized in several scientific investigations for characterizing forest ecosystems and changes over large spatial and temporal scales [50–53].

#### FIA data for the study

We used an Ivan damage boundary map from the Alabama Forestry Commission [47] to select FIA plots located inside the damage zone. The FIA data for the plots in the area were downloaded and extracted from the Microsoft Access State database applications (USDA Forest Service FIA Datamart webpage (https://apps.fs.usda.gov/fia/datamart/datamart.html) [45, 54]. We selected our sample from post Ivan plots that were collected between 2006–2012 (Post-Ivan_1 event). Based on plot conditions, these plots were classified into four categories: ND (No Disturbance; plots had no disturbance), NDBH (No Disturbance But Harvesting; plots had no natural disturbance but had harvesting done), ID (Ivan Disturbance; plots had damage caused by Hurricane Ivan) and IDAH (Ivan Disturbance and Harvesting; plots had damage caused by Hurricane Ivan and harvesting done). There were a relatively large number of plots for ND and NDBH, so we randomly selected a nearly equal number of plots from them (43 and 45, respectively) that were in proximity to ID and IDAH plots. ID and IDAH plot conditions were represented by 43 and 19 plots, respectively. Thus, a total of 150 plots, representing 4 different plot conditions were selected for Post-Ivan_1 event (Table 1). Of these plots, 130 plots were in Alabama and 20 were in Florida. These 150 plots were then tracked during the inventory periods of 2000–2003 (pre-Ivan condition), 2006–2012 (first post-Ivan condition), 2013–2018 (second post-Ivan condition). Therefore, in total, the study analyzed 450 plots collected during 2000–2018. We called these inventory periods Pre-Ivan, Post-Ivan_1 and Post-Ivan_2 and represented them by their approximate mid years as 2002, 2009, and 2016, respectively (Table 2). Thus, effectively, Pre-Ivan and Post-Ivan_2 plots were tracked and analyzed for each group of Post-Ivan_1 plots.

**Table 1:** Description of disturbances in the study area.

Disturbance code	Disturbance definition	# of plots
ND	No Disturbance; plots had no natural disturbance and no harvesting	43
NDBH	No Disturbance But Harvesting; plots had no natural disturbance but had harvesting done	45
ID	Ivan Disturbance; plots had damage caused by Hurricane Ivan and were not harvested	43
IDAH	Ivan Disturbance And Harvesting; plots had damage caused by Hurricane Ivan and were harvested	19
		150 (Total)

**Table 2:** Sampling intervals for the FIA plots used in the study.

	Plot inventory period	Approximate mid-year of inventory period	# of FIA plots
Pre-Ivan	2000–2003	2002	150
Post-Ivan_1	2006–2012	2009	150
Post-Ivan_2	2013–2018	2016	150
Total			450

FIA classifies the constituent species of the plots into seedlings, saplings, and trees based on their sizes and collects specific data about them. Information accessed about the plots included plot location (geographic coordinates), species, dbh and total height of all trees and saplings, and count data of seedlings for the three inventory periods. We also noted the FIA forest type group and stand origin (natural or artificial regeneration) information associated with each plot.

### Data analyses

For all 450 plots, we calculated (1) percentage of dead, live, and removed trees and saplings (2) quadratic mean diameter (QMD) (3) basal area (4) density of trees, saplings, and seedlings, (5) dry aboveground biomass (AGB) of live trees, (6) mean height of live trees, and the following additional attributes separately for the tree layer, sapling layer, and/or seedling layer: (7) species richness, (8) Shannon diversity index, (9) species importance value percent (IVP), and (10) basal area by species. From the values of these variables in individual plots we obtained their means and standard errors for each plot condition for each inventory period.

Among the above-mentioned variables, removed trees and saplings included all the trees and saplings that were removed in any harvest operation, including, commercial thinning, clearcut, partial, salvage, or seed-tree/shelterwood harvest. AGB, QMD, BA, tree density and height were calculated for trees with dbh≥12.7 cm. AGB was calculated using published species-specific biomass equations and adjustment factors for the tree components as per the FIA protocols [55, 56]. Species richness of tree layer, sapling layer, and seedling layer represented the number of species in these layers in each plot. The Shannon diversity index was calculated as -∑p_i_ ln p_i_, where “p_i_” is the proportion of basal area constituted by individuals of species “i” [57]. A higher value of Shannon diversity index represented a more diverse plot with more evenly distributed constituent species. IVP of each species of saplings and trees was calculated as an average of relative density percent, relative frequency percent, and relative dominance percent that was used to rank the dominance of species in a plot condition group (i.e. for each of ND, NDBH, ID and IDAH group) [58]. Since FIA records only count data for seedlings, IVP for seedling species represented only relative density percent of the species in each group.

Data were analyzed using IBM SPSS 21 and SAS 9.3 statistical packages. SAS PROC mixed procedure with a Tukey multiplicity adjustment was used for pairwise comparison of the means at an alpha level (α) of 0.05. Standalone PC-ORD Version 6 software was used for computing species richness, Shannon’s diversity index, species IVP and tree community analyses [58].

## Results

### Structural changes

#### Dead, live, and removed tree ratios

**Fig 2:**
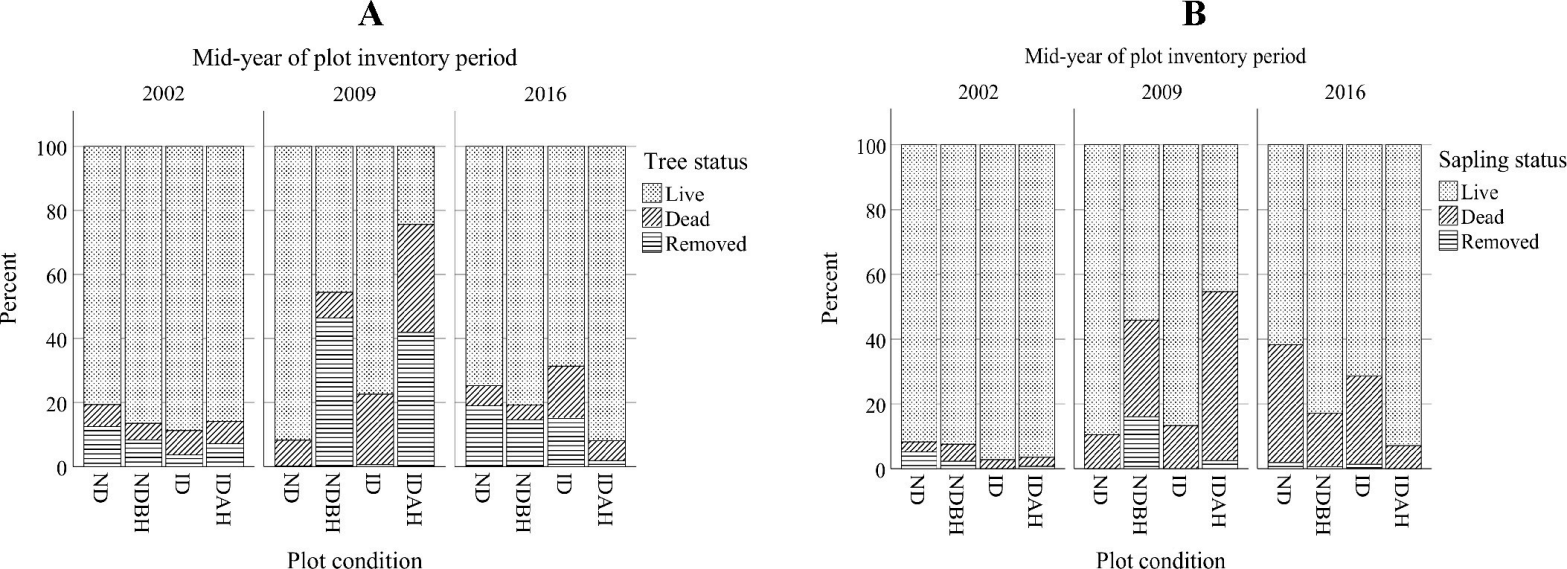
Percent of live, dead and removed trees (A) and saplings (B) across different plot conditions by mid-year of plot inventory period. Plot conditions are represented by ND (no disturbance), NDBH (no natural disturbance but harvested) ID (Ivan damaged) and IDAH (Ivan damaged and harvested).

All plot conditions showed an increased percent of dead trees and saplings in 2009 following the hurricane (Fig 2A and 2B). However, ID and IDAH plots, not surprisingly, had a much greater percent of dead trees (22% and 33.7%, respectively) than the ND and NDBH plots (7.9% and 8%, respectively) (Fig 2A). IDAH and NDBH had a greater percentage of removed trees as well (41.9% and 46.4%, respectively). The trees removed in IDAH plots in 2009 following Hurricane Ivan represented salvage harvest on 22% of the plots while remaining plots were either clear cut or partially harvested. The high percent of dead saplings in NDBH plots (29.8%) in 2009 is likely because of the residual damage caused by the harvests (Fig 2B). Similarly, the even greater percent of dead saplings in IDAH plots (52.2%) in 2009 is likely because of the hurricane damage and residual damage caused by the harvests. Harvest in IDAH plots during 2009 resulted in high percentages of live trees and saplings (92% and 92.8%, respectively) in 2016. By 2016, the differences among the plot types had diminished, though the percent of dead saplings was somewhat higher compared to 2002 (Fig 2B).

#### Quadartic mean diameter, basal area and density

**Fig 3:**
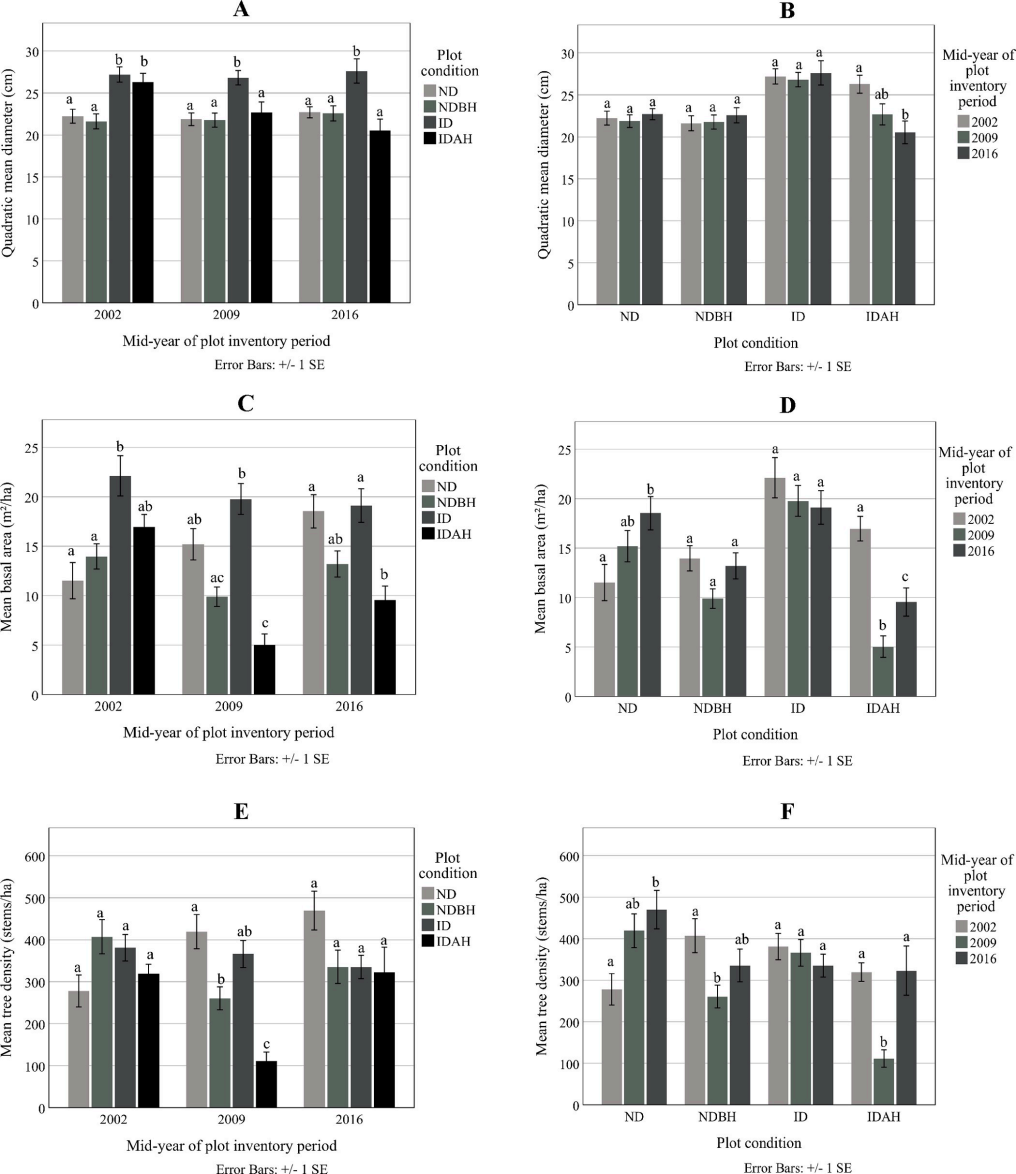
Quadratic mean diameter, mean basal area, stems per ha of trees at the mid-year plot inventory period by plot condition (A), (C) and (E), and by plot inventory period (B), (D) and (F). Plot conditions are represented by ND (no disturbance), NDBH (no natural disturbance but harvested) ID (Ivan damaged) and IDAH (Ivan damaged and harvested). Means with the same letter were not significantly different (P>0.05) using Tukey–Kramer multiple pairwise mean comparisons.

Prior to the hurricane, the affected ID and IDAH plots had greater QMD (27.2 cm and 26.3 cm, respectively) and mean basal area (22.1 m^2^ ha^-1^ and 16.9 m^2^ ha^-1^, respectively) than those of ND (22.2 cm and 21.6 m^2^ ha^-1^) and NDBH plots (11.5 cm and 14 m^2^ ha^-1^) (Fig 3A–3D). However, tree density in all plot conditions was similar (Fig 3E) and ranged between 278 to 407 stems ha^-1^.

#### Dry aboveground biomass and height

**Fig 4:**
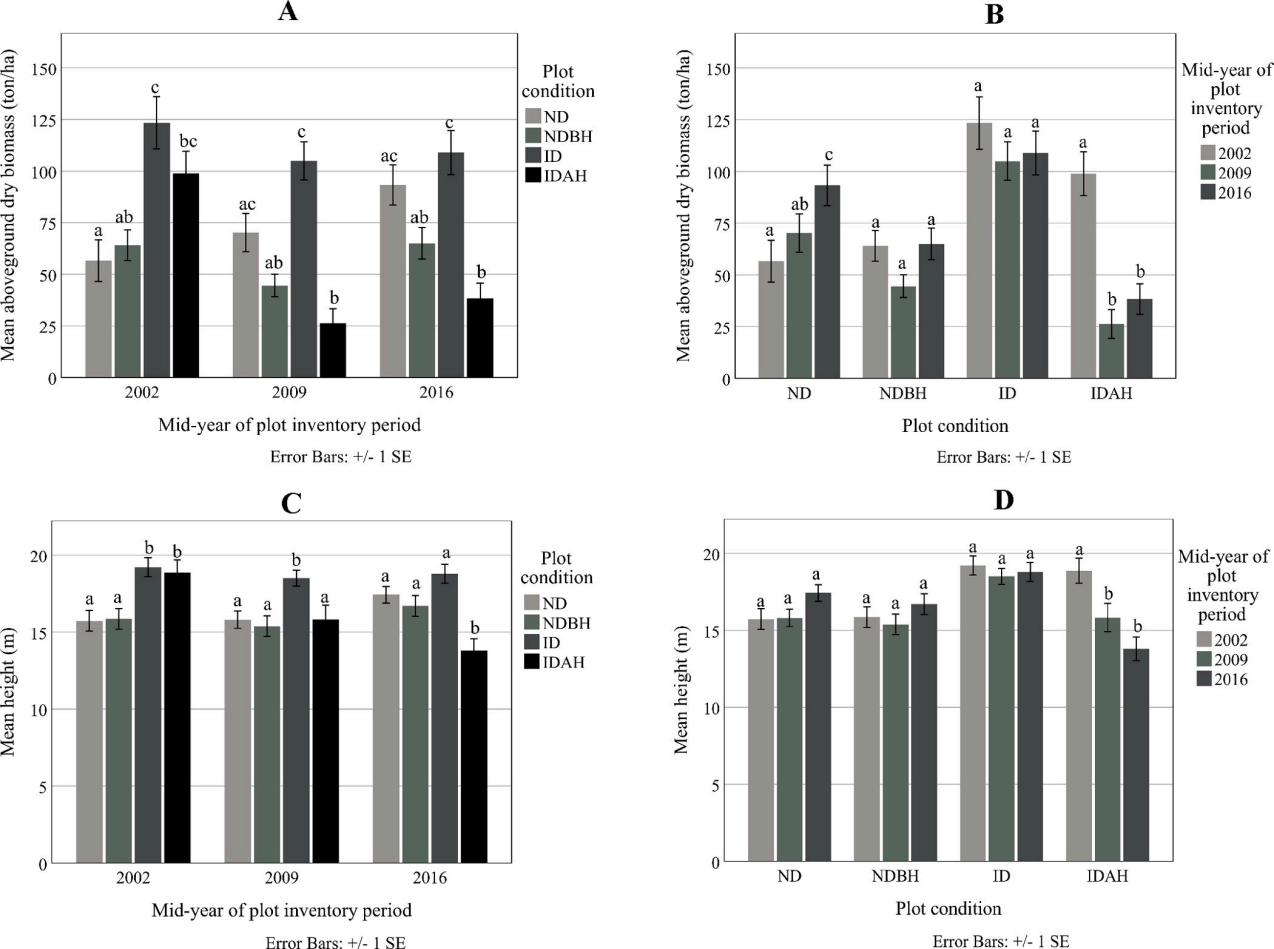
Mean aboveground dry biomass and mean height of trees at the mid-year plot inventory period by plot condition (A) and (C) and mean aboveground dry biomass and mean height for the plot condition by mid-year plot inventory period (B) and (D). Plot conditions are represented by ND (no disturbance), NDBH (no natural disturbance but harvested) ID (Ivan damaged) and IDAH (Ivan damaged and harvested). Means with the same letter were not significantly different from each other (P>0.05) using Tukey–Kramer multiple pairwise mean comparisons.

The affected plots (ID and IDAH) had greater aboveground dry biomass (123.4 ton ha^-1^and 98.9 ton ha^-1^, respectively) and mean tree height (19.2 m and 18.9 m, respectively) than ND (56.7 ton ha^-1^ and 15.7 m) and NDBH plots (64.1 ton ha^-1^ and 15.9 m) prior to the hurricane in 2002 (Fig 4A–4D).

Following the hurricane, the aboveground biomass in the ID plots decreased to the level of the ND plots. Biomass in the IDAH plots was the lowest (26.3 ton ha^-1^) because of the extensive hurricane damage and harvest. These differences in the biomass following hurricane persisted until 2016. Similar observations were made for mean height (Fig 4C and 4D).

Within the plot conditions, as expected, biomass of the ND plots increased consistently over 14 years (Fig 4B). ID plots showed no change in biomass while IDAH plots had lower biomass as compared to pre-hurricane conditions. Mean height significantly decreased from 18.9 m in 2009 to 13.8 m in 2016 only in the IDAH plots, highlighting the effect of harvesting on this plot conditions structure (Fig 4D).

#### Sapling and seedling densities

**Fig 5:**
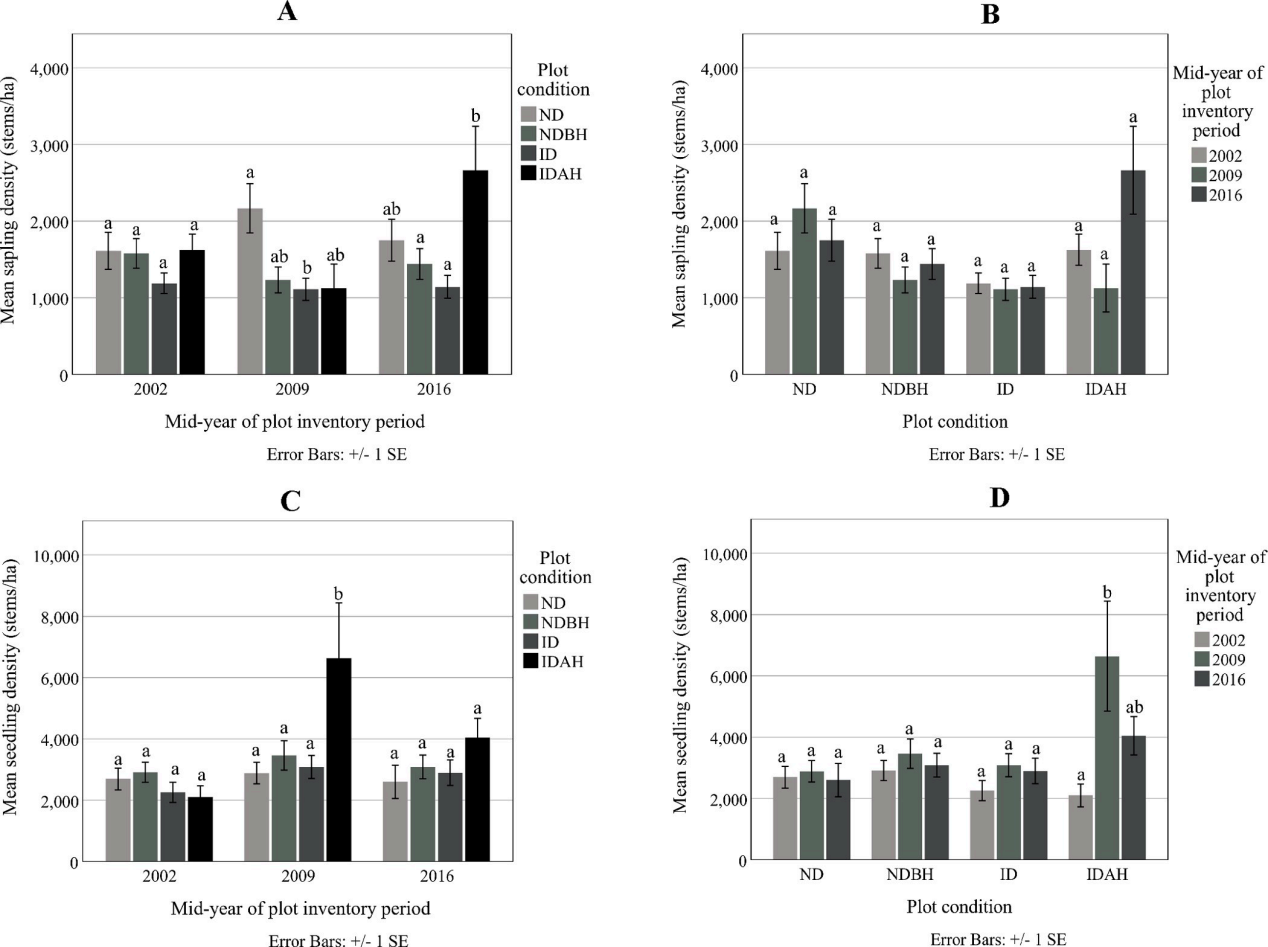
Mean sapling density and seedling density at the mid-year plot inventory period by plot condition (A) and (C), and mean sapling density and seedling density for the plot condition by mid-year plot inventory period (B) and (D). Plot conditions are represented by ND (no disturbance), NDBH (no natural disturbance but harvested) ID (Ivan damaged) and IDAH (Ivan damaged and harvested). Means with the same letter were not significantly different from each other (P>0.05) using Tukey–Kramer multiple pairwise mean comparisons.

Sapling and seedling densities were similar in all four plot conditions before the hurricane (Fig 5A–5D). Post-hurricane, however, sapling density in ID plots (1111 stems ha^-1^) was significantly lower than in the ND plots (2167 stems ha^-1^) in 2009 (Fig 5A). But by 2016, sapling density in ID plots had reached statistically similar densities as ND plots. The hurricane did not appear to have affected seedling density in the ID plots compared in 2009 or 2016, as it was similar to the density in the ND plots (Fig 5C). The IDAH plots had the greatest seedling density in 2009, which was because of the newly established plantations (on 32% of the plots) or natural regeneration following harvest or hurricane damage. By 2016, seedling density was similar among the plot conditions.

The IDAH plots, which had the highest seedling density (6639 stems ha^-1^) in 2009, also had the highest sapling density (2665 stems ha^-1^) in 2016 (Fig 5A and 5C), as seedlings had grown to sapling size. While there were some significant differences in seedling and sapling densities among the plot conditions, interestingly, the sapling density within a plot condition did not change significantly from before to after the hurricane. Seedling density within plot conditions also did not change pre- and post-hurricane, except in IDAH plots where it increased in 2009, as noted above.

### Compositional changes

#### Species richness and diversity (trees, saplings, seedlings)

Prior to the hurricane in 2002, ID and IDAH plots had higher species richness (6.8 and 7.2, respectively) and Shannon diversity index (1.32 and 1.18, respectively) in tree layer compared to ND and NDAH plots (Tables 3 and S1). However, ND and NDAH plots had higher species richness and Shannon diversity index in the seedling layer than ID and IDAH plots. Sapling species richness and diversity varied among the four plot conditions. After the hurricane, in 2009, there was a decrease in species richness of trees and saplings, but an increase in seedling species richness in the ID plots. After 2009, however, species richness of trees and saplings increased and either equaled or exceeded the pre-hurricane levels (Table 3). In contrast, species richness in ND plots increased for trees, but first increased and then decreased for seedling and saplings. Shannon diversity index exhibited similar pattern as species richness (Table 3). The IDAH plots, however, showed somewhat different responses, notably in the seedling species and diversity responses. Here, the seedling diversity and richness continued to increase following the hurricane.

**Table 3:** Species richness and Shannon’s diversity index of different plot conditions by mid-year of plot inventory period. Minimum and maximum values are in parenthesis.

		Tree			Sapling			Seedling		
Plot condition*	Mid-year of plot inventory period	Plots	Mean species richness	Mean Shannon's diversity index	Plots	Mean species richness	Mean Shannon's diversity index	Plots	Mean species richness	Mean Shannon's diversity index
ND	2002	36	4.6 (1, 13)	0.94 (0, 2.03)	40	3.6 (1, 10)	0.76 (0, 2.03)	42	4.7 (1, 9)	1.18 (0, 1.98)
	2009	41	4.8 (1, 13)	0.92 (0, 2.25)	40	4.0 (1, 12)	0.92 (0, 2.08)	40	4.8 (1, 13)	1.17 (0, 2.27)
	2016	39	5.0 (1, 14)	0.94 (0, 2.15)	35	3.7 (1, 10)	0.87 (0, 1.90)	41	4 (1, 12)	1.01 (0, 2.03)
NDBH	2002	41	3.9 (1, 11)	0.68 (0, 2.17)	38	3.1 (1, 8)	0.75 (0, 1.75)	45	4.5 (1, 14)	1.07 (0, 2.45)
	2009	37	3.1 (1, 10)	0.58 (0, 2.05)	32	2.6 (1, 8)	0.58 (0, 1.60)	42	4.7 (1, 13)	1.11 (0, 2.3)
	2016	43	3.4 (1, 13)	0.56 (0, 2.16)	38	2.8 (1, 8)	0.60 (0, 1.61)	44	4.8 (1, 15)	1.1 (0, 2.23)
ID	2002	42	6.8 (1, 15)	1.32 (0, 2.25)	36	2.9 (1, 7)	0.66 (0, 1.61)	36	4.1 (1, 10)	1.06 (0, 2.07)
	2009	43	6.4 (1, 12)	1.30 (0, 2.01)	38	2.7 (1, 6)	0.65 (0, 1.72)	38	4.2 (1, 9)	1.02 (0, 2.02)
	2016	40	6.6 (1, 14)	1.32 (0, 2.09)	34	2.9 (1, 7)	0.67 (0, 1.59)	36	4.5 (1, 11)	1.07 (0, 2.04)
IDAH	2002	18	7.2 (1, 16)	1.18 (0, 2.25)	15	3.5 (1, 5)	0.83 (0, 1.51)	18	4.1 (2, 7)	1.18 (0.5, 1.82)
	2009	16	3.8 (1, 9)	0.90 (0, 1.70)	12	2.4 (1, 6)	0.56 (0, 1.61)	19	6.2 (1, 12)	1.27 (0, 2.12)
	2016	18	4.4 (1, 11)	0.93 (0, 2.07)	18	3.8 (1, 8)	0.81 (0, 1.96)	17	6.6 (1, 14)	1.39 (0, 2.41)

* Plot conditions are represented by ND (no disturbance), NDBH (no natural disturbance but harvested) ID (Ivan damaged) and IDAH (Ivan damaged and harvested).

#### Importance values (tree layer)

**Fig 6:**
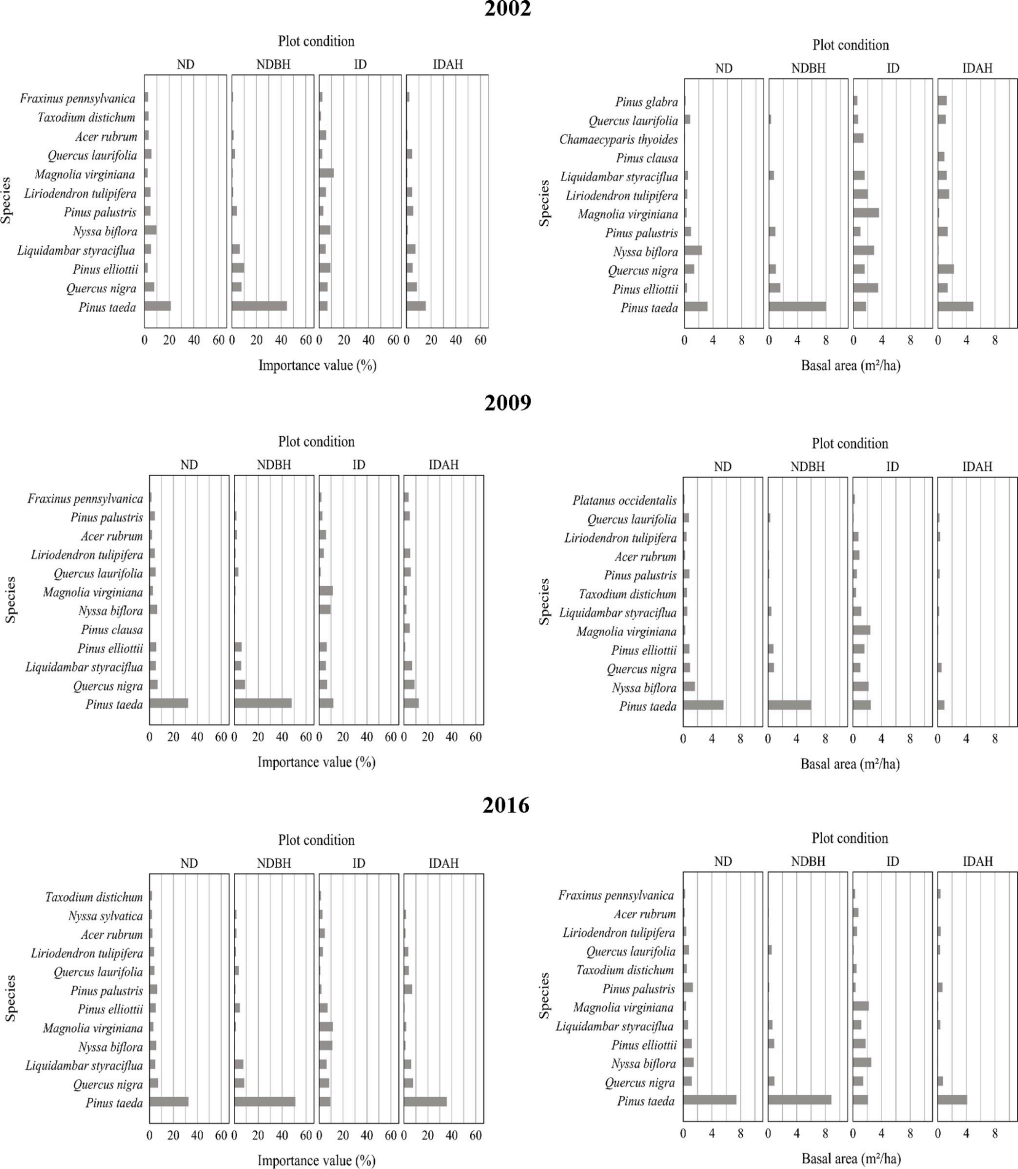
Importance value percent and basal area per hectare of species in the tree layer across different plot conditions by mid-year of plot inventory period. Plot conditions are represented by ND (no disturbance), NDBH (no natural disturbance but harvested) ID (Ivan damaged) and IDAH (Ivan damaged and harvested). See S2 Table for values of all species.

**Fig 7:**
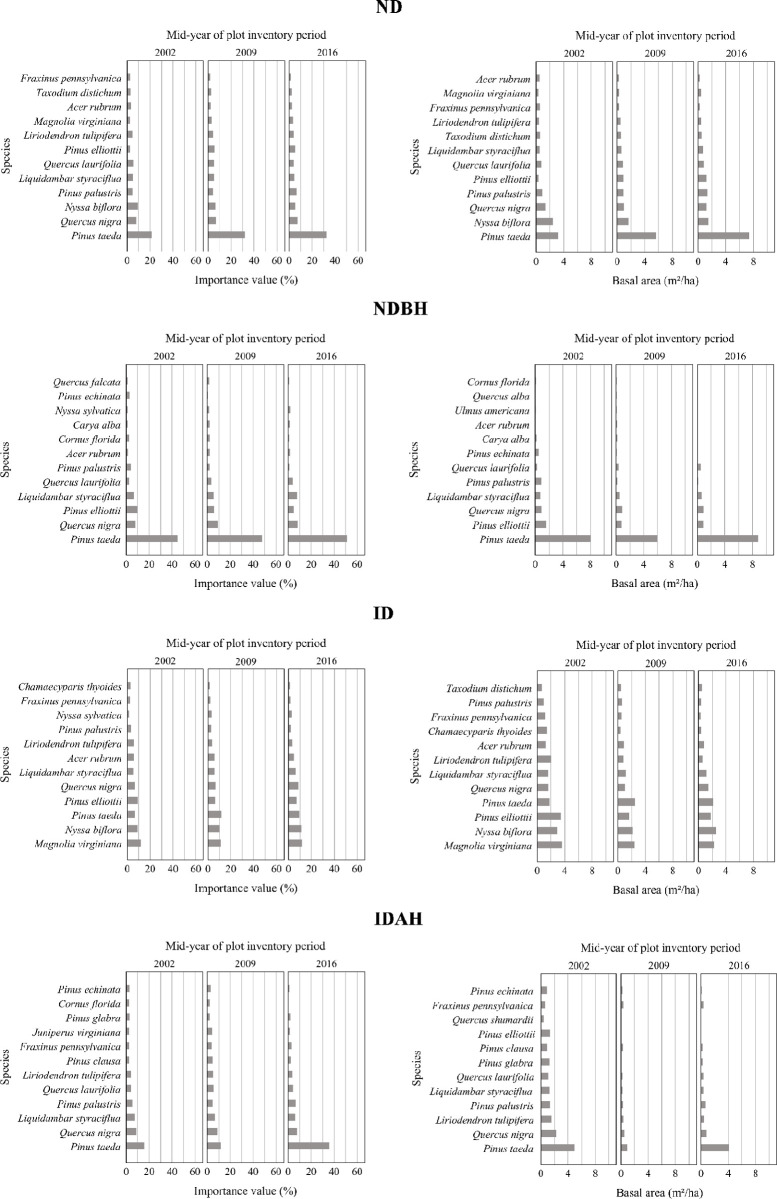
Importance value percent and basal area per hectare of species in tree layer across different mid-year of plot inventory period by plot condition. Plot conditions are represented by ND (no disturbance), NDBH (no natural disturbance but harvested) ID (Ivan damaged) and IDAH (Ivan damaged and harvested). See S2 Table for values of all species.

Different plot conditions had different IVP and basal area distribution profiles prior to the hurricane (Figs 6 and 7 and S2 Table), with relatively higher basal area proportion of sweetbay (*Magnolia virginiana*), tulip tree (*Liriodendron tulipifera*), swamp tupelo (*Nyssa biflora*) and sweetgum (*Liquidambar styraciflua*) in ID and IDAH plots (Fig 6). Post hurricane, not surprisingly, ND plots showed the least change (Fig 7). The hurricane disturbance in the ID plots influenced a large variety of the species present; however, it was most notable for slash pine, loblolly pine, sweetbay, tulip tree, and swamp tupelo. Most changes were observed in IDAH plots, including changes in the pine- dominated plots, including loblolly pine, longleaf pine, and slash pine. These represent dominant pine species (natural or planted) of the region, and were salvaged or harvested after the hurricane. In these plots, loblolly pine was the dominant species that was either replanted or regenerated naturally, followed by longleaf pine. Most of the less merchantable and less commercially important hardwood species, such as water oak (*Q*. *nigra*), shingle oak (*Q*. *latifolia*), tulip tree, and sweetgum apparently were left unharvested on the site following the hurricane, which then recovered slowly over the next several years. Loblolly pine, which had pre-hurricane IVP of 15.6% in IDAH plots, dominated this plot condition post hurricane with IVP of more than 35% in 2016 (Fig 7). In NDBH plots, loblolly pine always dominated pre- and post-hurricane with IVP between 44.7% and 51.3%, indicating it was the leading choice for managed forests in the region.

Overall, the ID plots had the most even species distribution, as judged by the IVP and basal area distributions of the different species (both hardwoods and pines) both pre- and post-hurricane, while all other plot conditions were highly skewed towards dominance by the commercial pine species. The greatest change over time in IVP and basal area was again observed in the IDAH plots where both hurricane and harvest disturbances had occurred.

#### Importance values (sapling layer)

**Fig 8:**
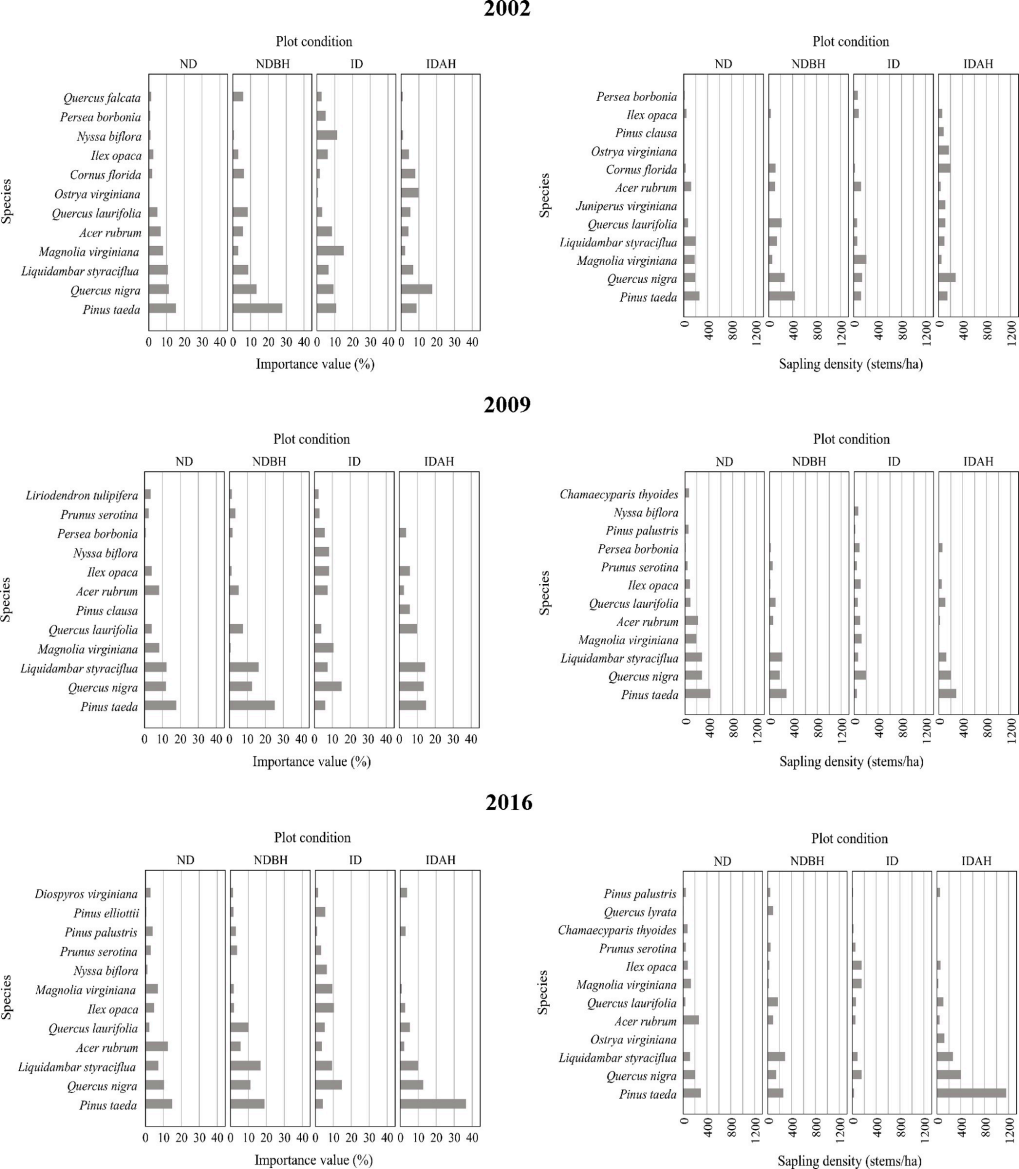
Importance value percent and stems per hectare of species in sapling layer across different plot conditions by mid-year of plot inventory period. Plot conditions are represented by ND (no disturbance), NDBH (no natural disturbance but harvested) ID (Ivan damaged) and IDAH (Ivan damaged and harvested). See S3 Table for values of all species.

**Fig 9:**
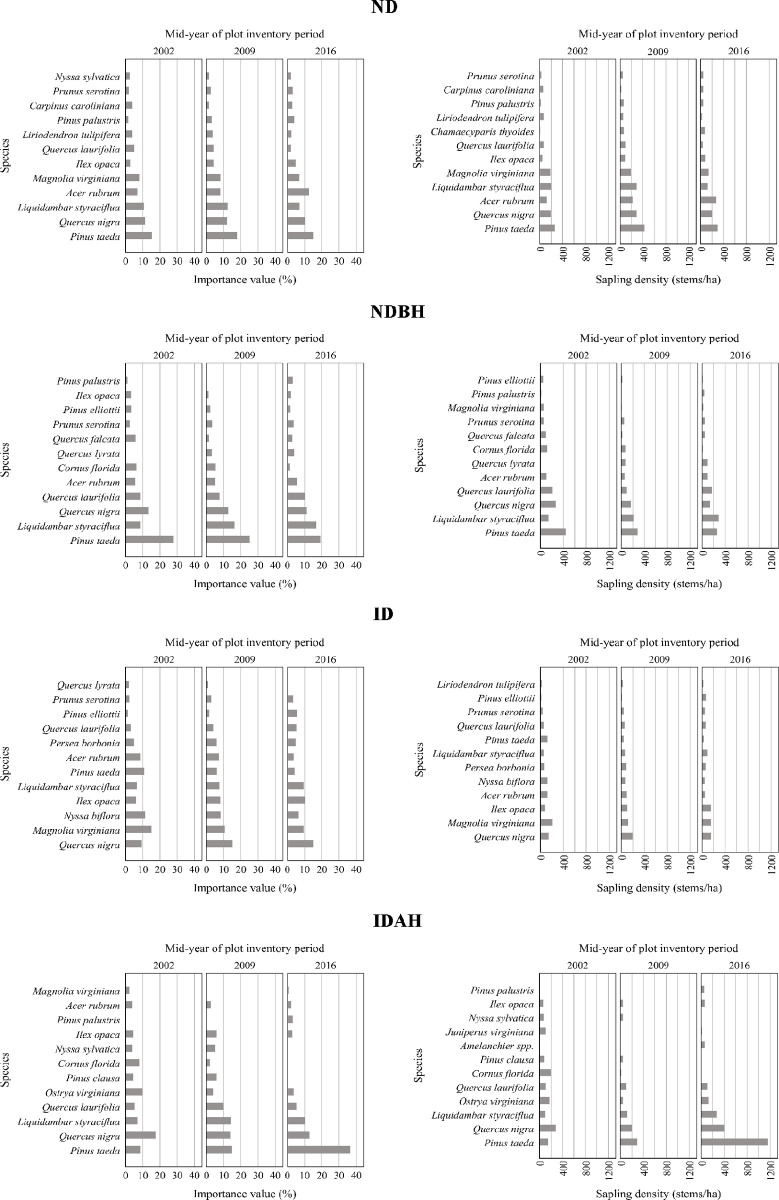
Importance value percent and stems per hectare of species in sapling layer across different mid-year of plot inventory period by plot condition. Plot conditions are represented by ND (no disturbance), NDBH (no natural disturbance but harvested) ID (Ivan damaged) and IDAH (Ivan damaged and harvested). See S3 Table for values of all species.

Figs 8 and 9 (and S3 Table) show the sapling IVP and basal area profiles for the four plot conditions, which varied prior as well as after the hurricane. Compared to the tree layer in ID and other plots, the sapling layer’s IVP and density was less affected during 2002 to 2016 (Figs 7 and 9). Least changes were observed in ND and NDAH plots (Fig 9). In ND plots, the changes in IVP and density observed between 2002 to 2009 and 2009 to 2016 of ND were due to natural mortality or recruitment of seedlings to saplings and saplings to tree sizes, respectively. Changes in NDBH and IDAH plots were influenced by harvesting operations. For example, it is evident that IVP and density of loblolly pine disproportionately increased in IDAH plots (from IVP of 8.6% in 2009 to 36.5% in 2016) because of planting (on 32% of the plots) or natural regeneration of this species following harvesting. In ID plots, apparently, saplings of loblolly pine and sweetbay were damaged more than those of other species. Slash pine also appeared to increase in IVP in ID plots from 1.1% in 2009 and to 5.6% in 2016 (Fig 9).

#### Importance values (seedling layer)

**Fig 10:**
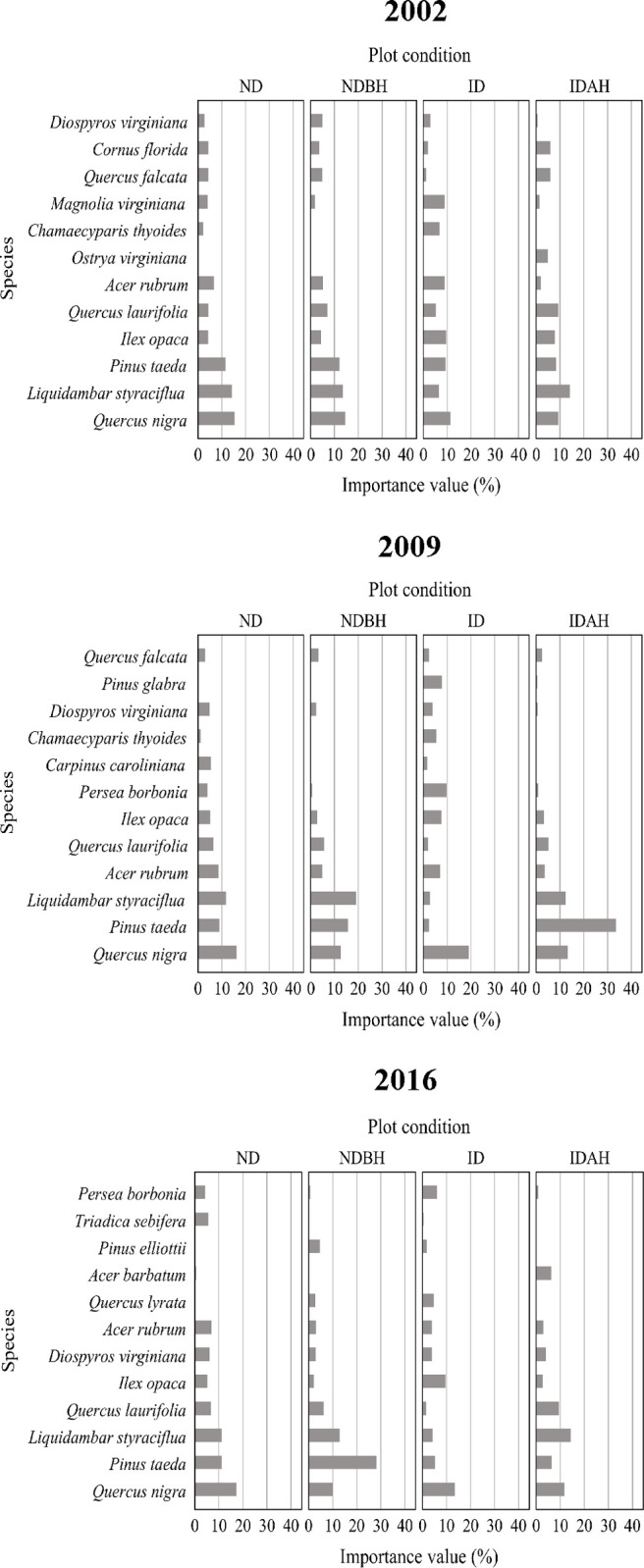
Importance value percent of species in the seedling layer across different plot conditions by mid-year of plot inventory period. Plot conditions are represented by ND (no disturbance), NDBH (no natural disturbance but harvested) ID (Ivan damaged) and IDAH (Ivan damaged and harvested). See S4 Table for values of all species.

**Fig 11:**
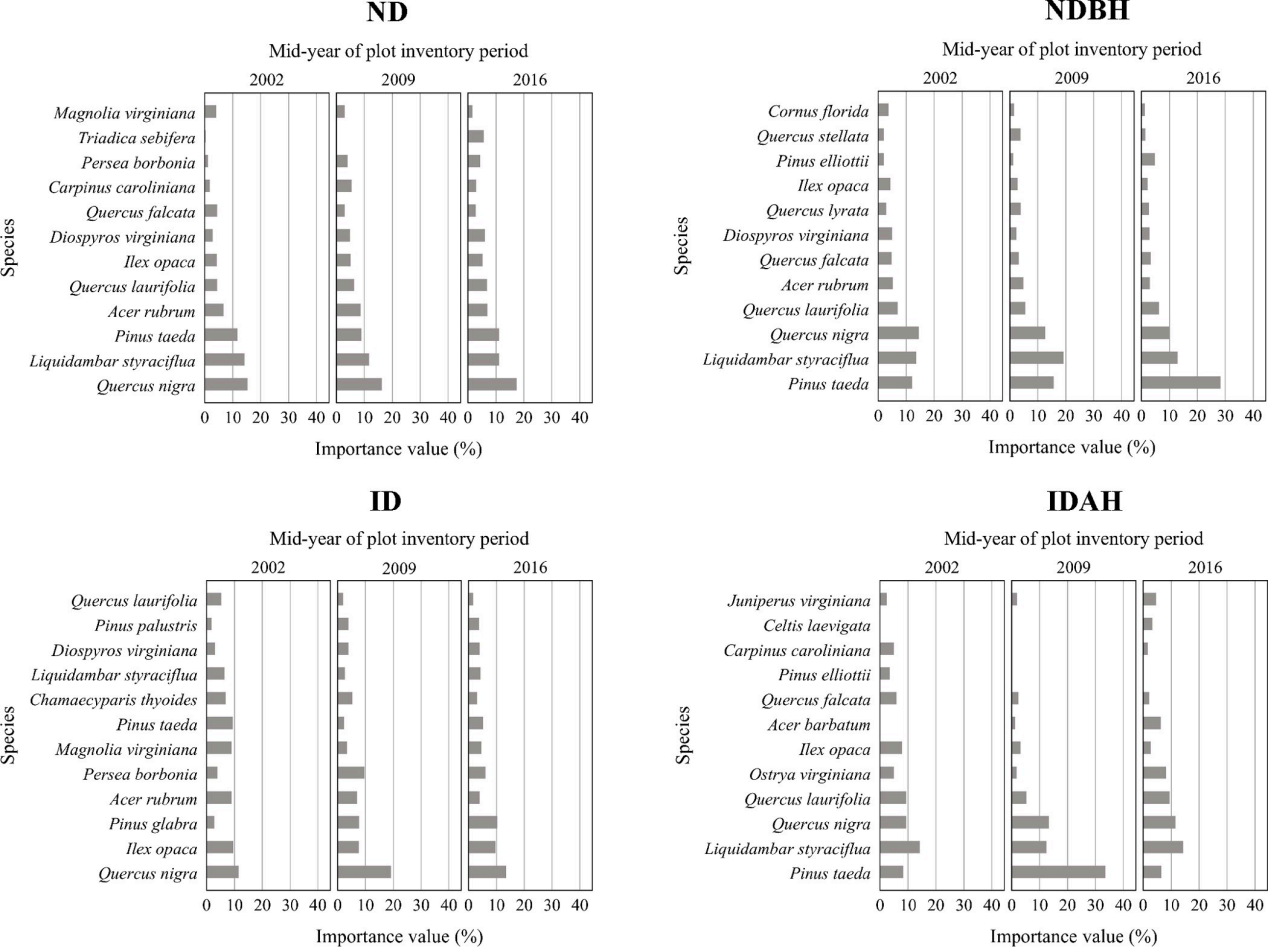
Importance value percent of species in the seedling layer across different mid-year of plot inventory period by plot condition. Plot conditions are represented by ND (no disturbance), NDBH (no natural disturbance but harvested) ID (Ivan damaged) and IDAH (Ivan damaged and harvested). See S4 Table for values of all species.

The seedling layer IVP profiles for the four plot conditions varied prior to as well as after the hurricane (Fig 10 and S4 Table). Water oak was among the dominant species (among five highest IVP) in the seedling layer among all plot conditions during all inventory periods (Fig 11). In ND plots, it was the species with the highest IVP during all inventory periods. Loblolly pine was the second most ubiquitous species in the seedling layer. Sweetgum was another one of the dominant species found in all plot conditions in all inventory periods, except the ID plots where it was not among the species with the highest five IVPs. In the ID plots, the five seedlings species with the highest IVP before hurricane were water oak (11.42%), American holly (*Ilex opaca*) (9.59%), loblolly pine (9.36%), red maple (*Acer rubrum*) (8.9%), and sweetbay (8.9%) (Fig 11). After hurricane, in 2009, redbay (*Persea borbonia*) (9.65%) and spruce pine (*P*. *glabra*) (7.75%) replaced loblolly and sweetbay from the top five species with highest IVPs, which indicates that these species responded naturally to the hurricane caused disturbance. These species maintained their dominance in ID plots in 2016 as well. The seedling species IVP distribution in ND and NDBH plots was not much affected during 2002–2016, except some increase in loblolly pine in IDAH plots. The greatest change was observed in IDAH plots.

### Plot characteristics and hurricane damage

The four plot conditions differed in pre-hurricane structure and composition, which significantly determined their resistance and response to the hurricane. Overall, as can be seen from Figs 2–11 and Table 3, the plots that were damaged by Hurricane Ivan (i.e., ID and IDAH plots) were characterized by (1) greater mean basal area, (2) greater QMD (3) greater mean tree height, (4) greater species richness and Shannon diversity index in the tree layer, (4) denser stocking of seedling and saplings, (5) lower proportion of dead trees or saplings, and (6) greater AGB. In contrast, however, the plots that were not damaged (ND and NDBH plots) usually were less stocked, had relatively smaller trees, but had similar density as the damaged plots.

## Discussion

Pre-hurricane stand characteristics were important factors that determined the vulnerability of the forest plots to hurricane damage. Plots with larger trees (diameter and height), greater basal area, and higher tree species diversity were disproportionately damaged by Hurricane Ivan. Individual species responses to hurricane differed, with loblolly pine’s importance considerably increasing following the hurricane. Overall, structure and composition of the affected plots were affected but nearly recovered to pre-hurricane conditions within 14 years after the hurricane.

### Forest structure and hurricane effects

Greater mortality among larger, older trees following windstorms is a common observation for a variety of forest types. Ojha et al. [59] reported that, during the period 1995–2018, hurricanes caused greatest damage in large diameter stands of major forest types in southeastern US, including loblolly–shortleaf pine, oak-gum-cypress and oak–hickory forest types. In southern pines, trees of larger size classes suffer greater damage and mortality following hurricanes [7, 60]. Taylor et al. [25] made similar observations in Acadian forests of Canada where taller and older trees were more vulnerable to windthrow. Younger, smaller trees have higher ratio of living sapwood to heartwood than older trees, which makes them elastic and flexible [61]. With age trees get taller resulting in a greater height-to-diameter (h:d) ratio, which lowers their stability and increases vulnerability to windthrow overturning [62, 63]. When large trees have attained their near maximum tree height, further growth is allocated to diameter increase, thus decreasing h:d ratio in very mature trees and potentially making them relatively more windfirm. Zampieri et al. [7] found some evidence that large-sized longleaf pine trees of mature age classes suffered lower mortality as compared to mid-sized trees of mature classes following Hurricane Michael in the Florida Panhandle. In addition to greater structural balance due to low h:d ratio, these older longleaf pine trees may also possess certain traits (e.g. a deeper taproot or higher wood density) that enabled them to survive thus far and helped develop greater resistance and resilience to high winds [64, 65].

Stand stocking effects on stand vulnerability to windstorms are more complex. Dense stands have a network of interlocking roots of adjacent trees that facilitate resistance to wind sway and movement. Dense canopy closure may help reduce wind drag over the canopy, thereby, elevating turbulence and eddies [66]. However, trees in high density stands tend to allocate more growth toward height and fine roots, which may make them more susceptible to uprooting or snapping during a windstorm, especially if the stand has been recently opened by thinning [24, 36]. Alternatively, open grown trees develop strong thick tapering stems with deep lateral roots that adapt them to conditions of continuous wind exposure [35]. Therefore, stand density effects will vary depending on past management, stage of stand development, species composition and age structure. These effects may also get modified by site conditions, especially in coastal areas. In Shelby Lakes near Gulf Shores in coastal Alabama, Bianchette et al., [17] reported that low elevations areas (< 3 m) suffered higher tree mortality (50–100% dead trees) than forested areas occurring on higher grounds (> 3 m elevation; < 50% tree mortality) following Hurricane Ivan. At this site, the damage pattern strongly suggested that saltwater intrusion and storm surge flooding, rather than wind damage, was the main cause for massive tree mortality.

While we found that the plots damaged by Hurricane Ivan had higher pre-hurricane QMD, basal area and aboveground biomass, and these plots also suffered greatest tree mortality; notably, these plots had recovered and attained similar levels of QMD, basal area and aboveground biomass by the next two sampling periods. This recovery was partially achieved by replanting commercial pines or natural regeneration of pines and other species and faster growth of residual stand following the hurricane. A study in Jamaica following Hurricane Gilbert showed that the average diameter growth rate of stems that survived the hurricane was greater than that of pre-hurricane for the 21-yr post-hurricane period leading to fast recovery of the forests [39]. In a similar instance in the southern US, Song et al. [43] reported that the species composition and dominance of most of the species had been recovered over a 10-year period following Hurricane Hugo.

### Forest composition and hurricane effects

Species have varying levels of resistance and resilience to windstorms due to differences in their canopy and root architecture, leaf shape and texture, and stem strength and elasticity [29]. The response of a species to windstorm also depends on its age, size, stand composition and diversity. Generally, species characterized with small, less dense crowns and flexible branches produce less wind drag, while the species with strong stem wood and deep rooting habit are less prone to stem breakage and uprooting [62, 63]. In Canada’s Acadian forests, Taylor et al. [25] found that while the taller stands were more vulnerable to windthrow, the effect varied with species composition. There, the stands dominated by spruce (*Picea* spp.) and balsam fir (*Abies balsamea*) suffered greater windstorm damage compared to hardwood and pine dominated stands. Observations from Australasia, Amazonia and other areas in the tropics show that species in fragmented forests were especially damaged from intense winds [38]. A study in the Caribbean mangroves forests found that the red mangrove (*Rhizophora mangle*) was significantly less resistant to hurricane damage than the black mangrove (*Avicennia germinans*) [40]. Among southern pine species, longleaf pine suffers lower mortality compared to other pine species, such as loblolly pine, slash pine or pond pine (*Pinus serotina*) when exposed to hurricane force winds [7, 30–33]. Among oaks, live oak (*Q*. *virginiana*) is less damaged than laurel oak (*Q*. *laurifolia*) and water oak following hurricanes [30, 43]. Tree species that commonly occur in the lower coastal plain (e.g., longleaf pine, bald cypress (*T*. *distichum*), and live oak) appear to suffer less damage than species with wider natural ranges [30, 43]. It is possible that the species that evolved within the southern coastal plain region developed structural or other attributes, possibly due to strong selection pressure from frequent exposure to high wind storms over their lifetime, that enabled them to be more resistant and resilient than those species whose evolutionary range extends beyond the coastal plain region [7, 30, 67]. We found in our study that the plots with greater diversity in tree layer were affected by the hurricane. This is in contrast to some other studies which found that mixed stands with high species diversity were more resistant to storm damages than less diverse stands (e.g., [68, 69]).

Varying species and stand level responses to hurricanes result in community changes. Vandermeer et al. [37] observed twofold to threefold increase in species richness in the damaged area during the 10 years after Hurricane Joan in Nicaragua. They suggested that large scale disturbances caused by hurricanes led to greater diversity in forest ecosystems than small scale tree fall created gaps and that periodic occurrences of large storms helped preserve diversity. In our study, ID plots observed a decrease in species richness of trees and saplings, but an increase in seedling species richness following the hurricane in 2009. This is understandable because new species emerged following hurricane would still be in seedlings stage by 2009, while the diversity lost in saplings and tree layers would still not be recovered until these seedlings grow to sapling or tree sizes. Following 2009, therefore, species richness of trees and saplings increased and either equaled or exceeded the pre-hurricane levels. In contrast, species richness in ND plots increased for trees, but first increased and then decreased for seedling and saplings. This is because the seedlings and saplings grew to tree sizes with no or relatively fewer new species regeneration during the period. Shannon diversity index exhibited similar pattern as species richness in our plots. The IDAH plots, however, showed somewhat different responses, notably in the seedling species and diversity responses. Here, the seedling diversity and richness continued to increase following the hurricane. This was possibly due to the reforestation and/or natural regeneration of several species on the hurricane affected sites that had been salvaged or harvested. In these plots, larger changes in sapling richness and diversity were also noted. These observations underlie the magnitude of effects that windstorm damages combined with salvage harvest operations can bring to regional forest ecosystems.

Loblolly pine was the dominant species in our plot conditions and its importance value increased following hurricane damage. This is not surprising. Loblolly pine is the commercially most important and common pine species of southern US. Following hurricane damage and harvestings done in some plot conditions, it was the choice species for regeneration. It is also a recognized pioneer species and responds well to the increase in site resources following canopy disturbances. Song et al. [43] also reported that the proportion of loblolly pine in the tree layer had increased following hurricane disturbance in the southern US.

Spruce pine and red bay showed massive recruitment (seedlings) in our hurricane damaged plots. Other studies also made similar observations where spruce pine massively recruited following large-scale disturbance caused by hurricanes (e.g. [70, 71]) to the extent that the age structures of spruce pine in mixed hardwood forest stands were found to consist of discrete age classes corresponding to previous dates of hurricanes experienced in the area [72]. Sweetbay was one of the most vulnerable species in our study as it lost basal area with no seedling or sapling increases, which are similar responses as observed by Batista and Platt [71]. Water oak showed low tree damage and was the dominant species in the seedling layer. American holly suffered low tree damage and increased in seedling and sapling layers. Very similar responses were observed by [71].

### Lessons for forest management

In a future driven by a changing climate, it is likely that windstorm events such as hurricanes and tornadoes will increase in strength and/or frequency, along with the other disturbance regimes, and their magnitudes may expand outside of the historical normal of Southern Coastal Plain [13–15]. If catastrophic wind damage is a contributor to the maintenance of tree species diversity in forests [37], the long-term effects of increasing frequency and intensity of storms could be consequential. In scenarios of high frequency of hurricanes in the southern US, it is possible that some tree species may only infrequently reach reproductively mature stage during the interval between storms, resulting in reduced prospects for maintaining persistent populations. Increasing frequency of storms, in such cases, could result in a reduction of species richness as species requiring longer time to reach reproductive maturity might disappear locally. Therefore, forest management plans need to account for large-scale stochastic mortality events and complex ecological effects of hurricanes to develop forest management approaches aimed at increasing forest resistance and resilience in order to effectively preserve critical southern forest ecosystems and biodiversity hotspot [7, 23].

Silvicultural management aimed at promoting windthrow resistant species and producing larger trees with lower h:d ratio (lower center of gravity) at an early age may help create tree and stand conditions resistant to catastrophic winds. Planting species such as longleaf pine at initial lower or moderate density may facilitate faster tree growth and a low h:d ratio earlier, that will lead to greater resistance to storm damage later [73]. Plantations raised at lower densities are exposed to more wind from an early age and invest resources more toward thickening of stem and of lateral roots [74]. Taylor et al. [25] made similar observations in Acadia forest where stands of <50% crown closure had the lowest risk of windthrow. Density management using precommercial thinning–which is currently an uncommon practice in southern pines—and woody control measures early in the stand development may also help develop long-term stand stability and resistance to storm damages [73]. Intensive silvicultural treatments that lead to faster tree growth, including fertilization and weed control and the use of suitable genetic deployment, may also result in less mortality and greater resistance to wind damage in young pine stands [75]. In addition, having some large-sized trees (e.g., in longleaf pine stands) of mature size classes may improve resistance to windstorm damage (or other disturbances, such as wildfires), and also resilience since these trees usually also serve as seed sources following disturbances [7]. The size threshold for longleaf pine or other species, however, need to be ascertained. Silvicultural practices—like thinning, improvement cuttings and timber stand improvement—that promote healthy stands of vigorous and strong trees with well-developed crowns and stems could help improve resistance and resilience of these forests over time. Such silvicultural practices provide several advantages, including, for example, (1) reducing stand stocking (2) regulating stand composition and structure towards resistant species, (3) faster increase in the diameter growth of retained trees leading to low tree h:d ratio, (4) increased tree crown size leading to spread of foliage and reduced wind drag, and (5) reduced fuel load. In the short term, however, a recently thinned stand may be susceptible to toppling and breakage from storms because of the lack of lateral root growth, high h:d ratio and reduced support between neighboring trees [36, 73, 76]. Several recently thinned pine stands were disproportionately damaged by Hurricane Michael in the Florida Panhandle (personal observations made by the authors). With time after thinning, however, the trees are expected to adjust their allometry (h:d ratio) to the new growing conditions and eventually the thinning is expected to have a positive effect. In US Southern Coastal Plain, the above-mentioned and also other suitable adaptive and management measures such as selection of planting site and species, stocking, and selection and timing of silvicultural treatments [77] should be encouraged in order to design and devise forest stands better able to withstand windstorm disturbances and their consequences.

The study has not tracked individual plots or trees in its analysis but presented an overall average and generalized response of the regional forest type groups to Hurricane Ivan over the entire impacted area and over the long-term. There are multiple factors that will affect these broad generalizations as some may be applicable only to individual plots or stands. All forest stands in a hurricane track are not subjected to equal wind speeds and differ to varying degrees in their meteorological, topographic, soil, and forest structural variables, that can influence vulnerability or damage caused to forest stands by windstorms. While wind speed and saltwater intrusion could be among the most influential variables affecting forest stands in coastal areas, the severity may vary according to stand structural variables and factors related to the site. Forest stands on well-drained and less rocky soils facilitate deeper rooting, growth and anchorage of trees and thus tend to be more resilient to windstorms. Similarly, stands composed of wind-resistant species would be less prone to windstorm damage because they possess traits that make them less susceptible to snapping and uprooting. This study has not investigated the mechanisms that explain the hurricane damages to forests but has described the actual effects observed over a period of 14 years following Hurricane Ivan.

## Conclusions

Our study made a number of important observations. Not all forest plots in the Hurricane Ivan track were affected. Some plots were affected more than others. Pre-hurricane stand structure determined the vulnerability of forest plots to hurricane damage. In general, the forest plots more prone to damage by the hurricane were characterized by (1) higher stocking, (2) larger sized trees, (3) more diverse tree species composition, (4) denser stocking of seedling and saplings, and (5) lower proportion of dead trees or saplings. After the hurricane, variable responses in structure and composition of the plots were observed. Regeneration of redbay and spruce pine dominated seedling layers in the hurricane affected plots. Loblolly pine was the species that increased most following the hurricane and dominated the hurricane affected area. Our study provides several lessons that could be used as a basis for resistance, resilience, and adaptation of southern US coastal forests, and of forests in other locations, to the increased incidents of windstorms and climate change.

Forest management will benefit from improved understanding of the mechanisms behind resistance and resilience of forest stands and species to catastrophic wind. Coastal forested ecosystems are also affected by flooding and salinity caused by storm events, which lead to expansion of tidal marshes into forested areas. Studies suggest that increase in sea level rise and increased incidents of storms will exacerbate salinity and storm surge in the southern coastal areas (e.g., [18]). Future studies should include water level and storm surge information (e.g., using watermark maps) in the affected areas to examine how flooding and salinity affect coastal forests and marsh migration into inland areas. Modeling studies should evaluate impacts of future storm surges, salinity and land-use-land-cover changes on coastal forests to inform forest management.

## Supporting information

S1 Table Number of species counted in seedling, sapling and tree layer for different plot conditions by mid-year of plot inventory period. (DOCX)

S2 Table Importance value percent of tree species (live, dbh≥12.7 cm) for each plot condition group by mid-year of plot inventory period. Dominance ranking order of the species for each group is presented in parenthesis. Top five dominant species are highlighted.
(DOCX)

S3 Table Importance value percent of sapling (live, 2.54≤dbh>12.7 cm) species for each plot condition group by mid-year of plot inventory period. Dominance ranking order of the species for each group is presented in parenthesis. Top five dominant species are highlighted.
(DOCX)

S4 Table Importance value percent of seedling (live, dbh<2.54 cm) species for each plot condition group by mid-year of plot inventory period. Dominance ranking order of the species for each group is presented in parenthesis. Top five dominant species are highlighted.
(DOCX)
