# Supplementary material for: Long-term effects of catastrophic wind on southern US coastal forests: Lessons from a major hurricane
Source: PLoS One. 2021 Jan 6;16(1):e0243362. doi: 10.1371/journal.pone.0243362 (PMC7787386; doi:10.1371/journal.pone.0243362)
Supplement: S1 Table — (DOCX) [file pone.0243362.s001.docx]

S1 Table: Number of species counted in seedling, sapling and tree layer for different plot conditions by mid-year of plot inventory period

|  |  | Seedlings | |  | Saplings | |  | Trees | |  |  |
| --- | --- | --- | --- | --- | --- | --- | --- | --- | --- | --- | --- |
| Plot condition* | Mid-year of plot inventory period | Plots | Species count |  | Plots | Species count |  | Plots | Species count |  |  |
| ND | 2002 | 42 | 41 |  | 40 | 39 |  | 36 | 42 |  |  |
|  | 2009 | 40 | 41 |  | 40 | 40 |  | 41 | 47 |  |  |
|  | 2016 | 41 | 42 |  | 35 | 38 |  | 39 | 48 |  |  |
| NDBH | 2002 | 45 | 40 |  | 38 | 26 |  | 41 | 41 |  |  |
|  | 2009 | 42 | 49 |  | 32 | 26 |  | 37 | 35 |  |  |
|  | 2016 | 44 | 43 |  | 38 | 30 |  | 43 | 41 |  |  |
| ID | 2002 | 36 | 39 |  | 36 | 29 |  | 42 | 56 |  |  |
|  | 2009 | 38 | 45 |  | 38 | 32 |  | 43 | 56 |  |  |
|  | 2016 | 36 | 42 |  | 34 | 30 |  | 40 | 57 |  |  |
| IDAH | 2002 | 18 | 32 |  | 15 | 27 |  | 18 | 50 |  |  |
|  | 2009 | 19 | 41 |  | 12 | 19 |  | 16 | 36 |  |  |
|  | 2016 | 17 | 41 |  | 18 | 30 |  | 18 | 38 |  |  |
|  | Total | 418 | **87** |  | 376 | **66** |  | 414 | **77** |  |  |

***** Plot conditions are represented by ND (no disturbance), NDBH (no natural disturbance but harvested) ID (Ivan damaged) and IDAH (Ivan damaged and harvested).
