# Supplementary material for: Long-term effects of catastrophic wind on southern US coastal forests: Lessons from a major hurricane
Source: PLoS One. 2021 Jan 6;16(1):e0243362. doi: 10.1371/journal.pone.0243362 (PMC7787386; doi:10.1371/journal.pone.0243362)
Supplement: S2 Table — Dominance ranking order of the species for each group is presented in parenthesis. Top five dominant species are highlighted. (DOCX) [file pone.0243362.s002.docx]

S2 Table: Importance value percent of tree species (live, dbh≥12.7 cm) for each plot condition group by mid-year of plot inventory period. Dominance ranking order of the species for each group is presented in parenthesis. Top five dominant species are highlighted.

|  |  |  |  |  |  |  | Mid-year of plot inventory period | | | |  |  |  |  |  |
| --- | --- | --- | --- | --- | --- | --- | --- | --- | --- | --- | --- | --- | --- | --- | --- |
|  |  | 2002 | | | |  | 2009 | | | |  | 2016 | | | |
| Scientific name | Common name | ND | NDBH | ID | IDAH |  | ND | NDBH | ID | IDAH |  | ND | NDBH | ID | IDAH |
| *Acer barbatum* | Florida maple |  |  | 0.2(48) | 1(27) |  | 0.2(41) |  |  | 1.7(20) |  | 0.2(38) |  | 0.2(56) | 1.7(14) |
| *Acer negundo* | boxelder |  |  |  |  |  |  |  | 0.2(55) |  |  |  |  | 0.2(45) | 0.6(36) |
| *Acer rubrum* | red maple | 3.5(8) | 1.5(10) | 5.8(6) | 0.8(31) |  | 2.2(11) | 2.3(6) | 5.8(6) |  |  | 2.4(10) | 1.7(7) | 4.8(7) | 1.1(19) |
| *Betula nigra* | river birch | 0.3(40) |  |  |  |  | 0.2(38) |  |  |  |  |  |  |  |  |
| *Carpinus caroliniana* | American hornbeam | 1.7(15) | 0.6(19) | 1.5(17) | 0.5(40) |  | 1.3(16) | 0.4(34) | 1.4(18) | 1.3(25) |  | 1.5(14) | 0.3(39) | 0.9(26) | 1(20) |
| *Carya alba* | mockernut hickory | 0.3(38) | 1.3(11) | 0.6(32) | 1(26) |  | 0.3(35) | 1.9(9) | 0.7(31) | 1.4(24) |  | 0.2(41) | 1.4(8) | 0.6(32) | 0.8(26) |
| *Carya aquatica* | water hickory |  | 0.3(34) | 0.4(38) |  |  | 0.2(39) |  | 0.2(48) |  |  | 0.2(36) | 0.3(28) | 0.5(35) |  |
| *Carya cordiformis* | bitternut hickory |  | 0.3(28) |  | 0.5(44) |  |  | 0.5(27) |  |  |  |  | 0.8(17) |  |  |
| *Carya glabra* | pignut hickory | 0.6(29) |  | 0.9(23) | 1(28) |  | 0.6(26) |  | 0.6(32) |  |  | 0.6(25) |  | 0.8(27) |  |
| *Carya illinoinensis* | pecan |  |  | 0.2(45) |  |  |  |  | 0.3(44) |  |  |  |  |  |  |
| *Carya ovata* | shagbark hickory |  |  | 0.2(47) | 1.2(21) |  |  |  | 0.2(47) | 1(31) |  |  |  | 0.2(43) | 0.6(32) |
| *Catalpa bignonioides* | southern catalpa | 0.3(41) | 0.3(35) |  | 0.7(35) |  |  | 0.4(32) |  | 1.5(22) |  |  | 0.3(36) |  | 0.6(31) |
| *Catalpa spp.* | catalpa spp. |  |  |  | 0.6(38) |  |  |  |  |  |  |  |  |  |  |
| *Celtis laevigata* | sugarberry | 1.5(19) | 0.4(23) | 0.7(30) |  |  | 0.5(29) | 0.4(29) | 1.1(22) |  |  | 0.4(29) | 0.3(29) | 0.9(25) | 0.6(38) |
| *Chamaecyparis thyoides* | Atlantic white-cedar | 0.5(34) |  | 2.9(10) |  |  | 0.5(28) |  | 1.4(16) |  |  | 0.7(21) |  | 1.3(20) |  |
| *Cornus florida* | flowering dogwood | 1(22) | 2.3(8) | 0.6(31) | 2.4(13) |  | 0.9(21) | 2(7) | 0.5(34) | 2.3(14) |  | 0.2(42) | 0.9(13) | 0.4(40) |  |
| *Crataegus spp.* | hawthorn spp. |  |  | 0.2(51) |  |  |  |  | 0.2(49) |  |  |  |  | 0.2(50) |  |
| *Diospyros virginiana* | common persimmon | 0.7(27) | 0.8(17) | 0.3(41) | 0.7(34) |  | 0.6(27) | 0.8(17) | 0.2(51) | 2(18) |  | 0.5(26) | 0.3(30) | 0.2(51) | 0.6(34) |
| *Fagus grandifolia* | American beech | 2.3(13) | 0.3(29) | 1(22) | 1.3(18) |  | 1(19) |  | 1.1(23) | 0.9(35) |  | 0.9(19) |  | 1.3(19) | 0.6(37) |
| *Fraxinus americana* | white ash |  |  |  | 1.3(20) |  |  |  |  |  |  |  | 0.6(23) |  |  |
| *Fraxinus pennsylvanica* | green ash | 3(10) | 0.8(16) | 2.6(11) | 2.4(12) |  | 1.7(14) | 0.7(20) | 2(11) | 3.8(9) |  | 1.4(16) | 0.8(15) | 1.6(16) | 2.8(7) |
| *Ilex opaca* | American holly | 1.5(17) | 0.3(31) | 0.8(27) | 1.4(17) |  | 1(17) | 0.5(25) | 1.4(17) | 2.4(12) |  | 1(18) | 0.7(19) | 2(10) | 2(9) |
| *Juglans nigra* | black walnut |  |  |  |  |  |  |  | 0.2(52) |  |  |  |  | 0.2(48) |  |
| *Juniperus virginiana* | southern redcedar | 0.5(32) | 0.5(21) | 0.3(44) | 2.5(11) |  | 0.7(24) | 0.7(18) | 0.4(38) | 4.6(8) |  | 0.5(28) | 0.6(24) | 0.4(39) | 1.3(17) |
| *Liquidambar styraciflua* | sweetgum | **5.2(5)** | **6.6(4)** | 5.3(8) | **7.3(3)** |  | **5.3(5)** | **5.8(4)** | 5.7(7) | **6.6(3)** |  | 4.8(6) | **7.6(3)** | 6.2(6) | **6(4)** |
| *Liriodendron tulipifera* | yellow-poplar | 4.8(7) | 1.1(15) | 5.6(7) | 4.5(6) |  | 4.3(7) | 1.1(14) | 3.9(8) | **5.2(5)** |  | 3.8(8) | 1.3(11) | 3.2(8) | 3.5(6) |
| *Magnolia acuminata* | cucumbertree |  |  |  |  |  |  |  |  |  |  |  | 0.3(40) |  |  |
| *Magnolia grandiflora* | southern magnolia | 0.4(35) | 0.3(37) | 1.2(19) |  |  | 1(18) | 0.4(35) | 1.9(12) |  |  | 1.5(15) | 0.3(32) | 1.8(14) |  |
| *Magnolia macrophylla* | bigleaf magnolia |  |  | 0.2(46) | 0.4(50) |  |  |  | 0.2(46) |  |  |  |  | 0.2(52) |  |
| *Magnolia tripetala* | umbrella magnolia |  |  |  |  |  |  |  |  |  |  |  |  | 0.2(53) |  |
| *Magnolia virginiana* | sweetbay | 2.6(12) | 0.6(20) | **12(1)** | 0.6(37) |  | 2.7(9) | 0.9(15) | **11.5(2)** | 2.3(13) |  | 3.3(9) | 1.3(9) | **11.6(1)** | 1.9(11) |
| *Melia azedarach* | chinaberry |  | 0.4(24) |  |  |  |  | 0.9(16) |  |  |  |  | 0.6(22) |  |  |
| *Morus rubra* | red mulberry |  | 0.3(38) | 0.5(34) | 0.5(41) |  | 0.2(45) |  | 0.4(36) |  |  | 0.2(43) |  | 0.7(30) |  |
| *Nyssa aquatica* | water tupelo | 1.1(21) |  | 0.5(35) |  |  | 0.4(33) |  | 0.5(35) |  |  | 0.3(34) |  | 0.2(44) |  |
| *Nyssa biflora* | swamp tupelo | **9.6(2)** | 0.3(25) | **9.2(3)** | 1.2(22) |  | **6.4(3)** | 0.4(30) | **9.7(3)** | 1.9(19) |  | **5.6(4)** | 0.3(41) | **11.1(2)** | 1.2(18) |
| *Nyssa sylvatica* | blackgum | 1.5(18) | 1.1(14) | 1.7(14) | 2(15) |  | 1.4(15) | 1.6(11) | 2.9(9) | 2.1(16) |  | 1.9(12) | 1.8(6) | 2.8(9) | 1.8(12) |
| *Ostrya virginiana* | eastern hophornbeam |  |  | 0.2(52) | 0.6(39) |  | 0.2(46) |  | 0.3(43) | 1.3(26) |  | 0.2(44) |  | 0.2(54) | 0.7(27) |
| *Oxydendrum arboreum* | sourwood | 0.5(33) | 0.3(30) | 0.3(43) | 0.7(33) |  | 0.3(37) | 0.5(28) | 0.4(40) |  |  | 0.3(35) | 0.3(37) | 0.4(37) |  |
| *Persea borbonia* | redbay |  |  | 1.2(20) |  |  |  |  | 1.3(19) | 0.9(36) |  | 0.2(45) |  | 1.1(22) | 0.7(28) |
| *Pinus clausa* | sand pine |  |  |  | 2.9(10) |  |  |  |  | 4.8(7) |  |  |  |  | 2.3(8) |
| *Pinus echinata* | shortleaf pine | 0.3(39) | 3.1(6) | 1.4(18) | 3.1(9) |  |  | 0.6(23) | 1.4(15) | 2.8(11) |  |  | 0.3(31) | 1.5(17) | 1(21) |
| *Pinus elliottii* | slash pine | 2.7(11) | **9.9(2)** | **9.2(2)** | **5.1(5)** |  | **5.6(4)** | **6.1(3)** | **6.4(5)** | 1(30) |  | **5.2(5)** | **4.8(4)** | **7(5)** | 0.6(33) |
| *Pinus glabra* | spruce pine | 0.9(24) |  | 1.8(13) | 3.2(8) |  | 0.4(30) |  | 1.6(13) | 2.1(17) |  | 0.2(37) | 0.3(38) | 1.9(12) | 1.9(10) |
| *Pinus palustris* | longleaf pine | 4.9(6) | **4.2(5)** | 3.3(9) | **5.5(4)** |  | 4.3(8) | 1.9(8) | 2.6(10) | 4.9(6) |  | **6.5(3)** | 1.1(12) | 1.9(11) | **6.6(3)** |
| *Pinus taeda* | loblolly pine | **21.2(1)** | **44.7(1)** | **6.6(5)** | **15.6(1)** |  | **32.3(1)** | **48(1)** | **11.9(1)** | **12.2(1)** |  | **32.5(1)** | **51.3(1)** | **9.3(3)** | **35.7(1)** |
| *Pinus virginiana* | Virginia pine |  |  |  |  |  |  |  |  |  |  |  | 0.3(34) |  |  |
| *Planera aquatica* | water-elm, planertree |  |  |  |  |  | 0.2(42) |  |  |  |  | 0.2(46) |  |  |  |
| *Platanus occidentalis* | American sycamore | 0.7(28) | 0.3(39) | 0.6(33) | 1(24) |  | 0.9(20) |  | 0.8(28) |  |  | 0.9(20) |  | 0.6(31) |  |
| *Prunus serotina* | black cherry | 0.6(30) | 1.5(9) | 0.4(39) | 0.4(48) |  | 0.4(31) | 0.7(21) | 0.8(29) |  |  | 0.4(32) | 1.3(10) | 0.7(29) |  |
| *Quercus alba* | white oak | 2.2(14) | 0.5(22) | 0.9(26) | 1.2(23) |  | 1.9(12) | 1.5(13) | 0.9(25) | 1.5(21) |  | 1.8(13) | 0.7(18) | 1.4(18) | 0.9(24) |
| *Quercus falcata* | southern red oak | 1.7(16) | 1.2(12) | 0.7(29) | 1.8(16) |  | 1.7(13) | 1.7(10) | 1(24) | 1(28) |  | 1.4(17) | 0.9(14) | 1.1(21) | 1.6(15) |
| *Quercus incana* | bluejack oak |  | 0.3(36) |  |  |  |  |  |  |  |  |  |  |  |  |
| *Quercus laevis* | turkey oak |  | 0.3(32) |  | 0.5(43) |  |  |  |  | 1(32) |  |  |  |  | 0.6(35) |
| *Quercus laurifolia* | laurel oak | **5.5(4)** | 2.6(7) | 2.4(12) | 4.4(7) |  | 5.1(6) | **3.4(5)** | 1.2(21) | **5.7(4)** |  | 4.1(7) | **3.9(5)** | 0.9(24) | **4(5)** |
| *Quercus lyrata* | overcup oak | 0.3(37) | 0.3(27) | 1.6(15) |  |  | 0.3(34) | 0.5(26) | 1.6(14) |  |  | 0.4(33) | 0.8(16) | 1.9(13) |  |
| *Quercus marilandica* | blackjack oak |  |  |  | 0.9(29) |  |  |  |  |  |  |  |  | 0.2(57) |  |
| *Quercus michauxii* | swamp chestnut oak | 0.8(26) |  | 0.2(50) | 0.8(32) |  | 0.6(25) |  | 0.2(45) | 1(29) |  | 0.6(24) |  |  | 0.9(22) |
| *Quercus muehlenbergii* | chinkapin oak |  |  |  | 0.4(49) |  |  |  |  |  |  |  |  |  |  |
| *Quercus nigra* | water oak | **7.8(3)** | **7.9(3)** | **6.7(4)** | **8.6(2)** |  | **6.7(2)** | **9(2)** | **6.7(4)** | **8.8(2)** |  | **7.2(2)** | **8.2(2)** | **8.5(4)** | **7.6(2)** |
| *Quercus pagoda* | cherrybark oak |  |  | 0.2(53) | 0.4(46) |  | 0.2(40) |  | 0.4(37) | 1.3(27) |  | 0.6(23) |  | 0.6(33) | 0.8(25) |
| *Quercus phellos* | willow oak | 1.2(20) |  | 1.1(21) | 0.4(47) |  | 0.3(36) |  | 0.9(26) |  |  |  | 0.7(20) | 1(23) |  |
| *Quercus rubra* | northern red oak |  |  |  |  |  |  |  |  |  |  |  |  | 0.2(47) |  |
| *Quercus shumardii* | Shumard oak |  |  | 0.3(40) | 1(25) |  |  |  | 0.2(50) |  |  |  |  | 0.2(46) |  |
| *Quercus sinuata* | Durand oak |  |  |  | 0.6(36) |  | 0.2(43) |  |  | 1(33) |  | 0.2(39) |  |  | 0.7(29) |
| *Quercus stellata* | post oak | 0.6(31) | 1.2(13) | 0.3(42) | 0.5(42) |  | 0.2(47) | 1.6(12) | 0.6(33) |  |  |  | 0.6(25) | 0.8(28) |  |
| *Quercus texana* | Texas red oak |  | 0.3(26) |  |  |  |  | 0.6(24) |  |  |  |  | 0.5(27) |  |  |
| *Quercus velutina* | black oak |  | 0.3(33) |  | 0.9(30) |  |  | 0.4(31) |  | 0.9(34) |  |  | 0.3(33) |  | 0.7(30) |
| *Quercus virginiana* | live oak |  |  | 0.5(36) | 0.4(45) |  |  |  | 0.4(39) | 1.4(23) |  | 0.2(47) |  | 0.5(36) | 0.9(23) |
| *Salix nigra* | black willow |  |  |  |  |  |  |  |  |  |  | 0.2(48) |  |  |  |
| *Sassafras albidum* | sassafras |  | 0.3(40) | 0.2(54) |  |  |  |  | 0.2(53) |  |  | 0.7(22) |  | 0.2(49) |  |
| *Taxodium ascendens* | pondcypress | 0.9(23) |  | 0.7(28) |  |  | 0.8(22) |  | 0.9(27) |  |  | 0.5(27) |  | 0.3(41) |  |
| *Taxodium distichum* | baldcypress | 3.2(9) |  | 1.5(16) |  |  | 2.5(10) |  | 1.3(20) |  |  | 2.2(11) |  | 1.6(15) |  |
| *Tilia americana* | American basswood |  |  | 0.2(55) |  |  |  |  |  |  |  |  |  |  |  |
| *Triadica sebifera* | Chinese tallowtree |  |  | 0.9(25) |  |  |  | 0.4(33) | 0.8(30) |  |  |  | 0.3(35) | 0.6(34) |  |
| *Ulmus alata* | winged elm | 0.3(42) | 0.3(41) | 0.2(56) | 1.3(19) |  | 0.2(44) | 0.7(19) | 0.2(54) | 3(10) |  | 0.2(40) | 0.7(21) | 0.4(38) | 1.8(13) |
| *Ulmus americana* | American elm | 0.9(25) | 0.8(18) | 0.9(24) | 2.1(14) |  | 0.7(23) | 0.7(22) | 0.2(56) | 2.2(15) |  | 0.4(30) | 0.5(26) | 0.3(42) | 1.5(16) |
| *Ulmus rubra* | slippery elm | 0.4(36) |  | 0.5(37) |  |  | 0.4(32) |  | 0.4(41) |  |  | 0.4(31) |  | 0.2(55) |  |
| Importance Value Percent is calculated as the average of relative frequency percent, relative density percent, and relative dominance percent. | | | | | | | | | | | | | | | |
