# Supplementary material for: Long-term effects of catastrophic wind on southern US coastal forests: Lessons from a major hurricane
Source: PLoS One. 2021 Jan 6;16(1):e0243362. doi: 10.1371/journal.pone.0243362 (PMC7787386; doi:10.1371/journal.pone.0243362)
Supplement: S3 Table — Dominance ranking order of the species for each group is presented in parenthesis. Top five dominant species are highlighted. (DOCX) [file pone.0243362.s003.docx]

S3 Table: Importance value percent of sapling (live, 2.54≤dbh>12.7 cm) species for each plot condition group by mid-year of plot inventory period. Dominance ranking order of the species for each group is presented in parenthesis. Top five dominant species are highlighted.

|  |  | Mid-year of plot inventory period | | | | | | | | | | | | | |
| --- | --- | --- | --- | --- | --- | --- | --- | --- | --- | --- | --- | --- | --- | --- | --- |
|  |  | 2002 | | | |  | 2009 | | | |  | 2016 | | | |
| Scientific_name | Common_name | ND | NDBH | ID | IDAH |  | ND | NDBH | ID | IDAH |  | ND | NDBH | ID | IDAH |
| *Acer barbatum* | Florida maple |  |  |  |  |  |  |  |  | 3.3(11) |  |  |  |  | 1.1(16) |
| *Acer negundo* | boxelder |  |  | 0.7(22) |  |  |  |  | 1.2(19) |  |  |  |  | 0.6(26) |  |
| *Acer rubrum* | red maple | **6.8(5)** | 5.6(7) | **8.5(5)** | 4(10) |  | **8.1(5)** | 5.1(6) | 7.3(6) | 2.5(12) |  | **12.5(2)** | **5.7(5)** | 3.6(10) | 2.2(9) |
| *Amelanchier spp.* | serviceberry spp. |  |  |  |  |  |  |  |  |  |  |  |  |  | 1.7(10) |
| *Asimina triloba* | pawpaw |  |  |  |  |  |  |  |  |  |  |  | 0.5(28) |  |  |
| *Carpinus caroliniana* | American hornbeam, musclewood | 3.7(9) | 0.7(18) | 1.4(18) | 2.9(12) |  | 1.4(16) |  | 2(13) |  |  | 2.8(10) |  | 2.9(12) | 0.6(30) |
| *Carya alba* | mockernut hickory | 0.8(25) | 0.4(24) | 0.5(29) |  |  | 1.4(15) | 2.2(10) | 0.5(29) | 1.8(17) |  | 0.9(23) | 1.8(12) | 0.6(27) |  |
| *Carya cordiformis* | bitternut hickory |  |  |  |  |  |  | 0.6(25) |  | 1.9(15) |  |  | 1(20) |  |  |
| *Carya glabra* | pignut hickory |  | 0.4(26) |  | 1.4(17) |  | 0.5(30) |  |  |  |  | 0.7(27) |  |  |  |
| *Celtis laevigata* | sugarberry |  |  |  |  |  |  |  | 0.5(32) | 2.5(13) |  |  | 0.5(30) | 0.5(30) |  |
| *Cercis canadensis* | eastern redbud | 0.4(35) | 0.4(25) |  |  |  |  | 1(19) |  | 1.7(19) |  |  |  |  | 1(17) |
| *Chamaecyparis thyoides* | Atlantic white-cedar | 0.5(28) |  | 0.7(23) |  |  | 1.9(12) |  | 0.7(25) |  |  | 3.3(8) |  | 1.5(17) |  |
| *Cornus florida* | flowering dogwood | 1.9(14) | **6.2(5)** | 1.6(16) | **8(4)** |  | 1.4(14) | **5.3(5)** | 0.9(23) | 1.8(16) |  | 0.4(36) | 1.5(15) | 0.7(25) |  |
| *Crataegus spp.* | hawthorn spp. | 0.4(38) |  |  | 1(25) |  | 0.6(25) |  |  |  |  | 0.5(29) |  | 0.8(24) |  |
| *Diospyros virginiana* | common persimmon | 1.4(18) | 1.9(13) |  | 2(15) |  | 1.3(17) | 1.9(11) | 0.5(30) |  |  | 2.8(11) | 1.5(16) | 1.5(16) | **3.8(5)** |
| *Fagus grandifolia* | American beech |  | 0.8(17) | 0.6(26) |  |  |  |  | 0.7(26) |  |  |  |  | 1.4(18) | 0.8(22) |
| *Fraxinus americana* | white ash |  |  |  |  |  | 0.3(40) |  |  |  |  | 0.6(28) | 1.1(18) |  |  |
| *Fraxinus pennsylvanica* | green ash | 1.2(20) |  | 1.7(15) |  |  | 1.3(18) |  | 2.2(12) |  |  | 2(16) |  | 2(13) | 1.4(12) |
| *Ilex opaca* | American holly | 2.6(10) | 3.1(9) | 6.1(7) | 4.4(8) |  | 4.1(7) | 1.2(17) | **8.1(4)** | **5.9(5)** |  | 4.8(6) | 2(11) | **10.4(2)** | 2.8(8) |
| *Juglans nigra* | black walnut |  |  |  |  |  |  |  |  |  |  |  |  |  | 0.9(19) |
| *Juniperus virginiana* | southern redcedar |  |  |  | 4.4(9) |  | 0.3(38) | 0.6(24) |  |  |  | 1.2(21) | 0.5(29) |  | 0.7(27) |
| *Liquidambar styraciflua* | sweetgum | **10.7(3)** | **8.6(3)** | 6.4(6) | **6.8(5)** |  | **12.3(2)** | **16.3(2)** | **7.4(5)** | **14.3(2)** |  | **7.1(4)** | **16.9(2)** | **9.4(4)** | **10(3)** |
| *Liriodendron tulipifera* | yellow-poplar | 4.1(7) | 0.4(23) | 2.4(11) | 1(23) |  | 3.4(8) | 1.4(15) | 2.3(11) |  |  | 2.3(12) | 2.2(10) | 1.4(19) | 0.8(20) |
| *Magnolia grandiflora* | southern magnolia | 0.5(34) |  | 1.5(17) |  |  | 0.9(22) |  | 1.7(15) |  |  | 1.3(19) |  | 1.3(21) | 0.8(23) |
| *Magnolia macrophylla* | bigleaf magnolia | 0.5(29) |  |  |  |  |  |  |  |  |  |  |  |  |  |
| *Magnolia virginiana* | sweetbay | **8(4)** | 3(10) | **15.1(1)** | 2.1(13) |  | **8.1(4)** | 0.7(21) | **10.6(2)** |  |  | **6.9(5)** | 1.8(13) | **9.4(3)** | 0.8(21) |
| *Morus rubra* | red mulberry |  |  |  |  |  |  |  |  |  |  | 0.4(38) |  |  | 0.7(29) |
| *Nyssa aquatica* | water tupelo | 0.4(37) |  |  |  |  | 0.4(33) |  |  |  |  |  |  |  |  |
| *Nyssa biflora* | swamp tupelo | 1(22) | 0.5(22) | **11.3(2)** | 1(22) |  | 0.4(35) |  | **8.2(3)** |  |  | 1.1(22) |  | **6.5(5)** |  |
| *Nyssa sylvatica* | blackgum | 2.4(12) | 1.9(12) | 1(20) | 4(11) |  | 1.5(13) | 1.3(16) | 1(20) | 5.1(7) |  | 2(15) | 1(21) | 1.1(22) |  |
| *Ostrya virginiana* | eastern hophornbeam |  |  | 0.5(27) | **9.8(2)** |  |  |  | 0.6(28) | 3.7(9) |  |  |  |  | 3.6(6) |
| *Oxydendrum arboreum* | sourwood | 2.4(11) | 0.5(21) |  | 0.9(27) |  | 0.3(36) | 0.6(23) |  |  |  | 0.4(35) |  | 0.6(28) |  |
| *Persea borbonia* | redbay | 0.9(24) |  | 4.9(8) |  |  | 0.7(24) | 1.7(12) | 5.9(8) | 3.8(8) |  | 0.9(24) |  | 4.8(8) | 1.2(13) |
| *Pinus clausa* | sand pine |  |  |  | 4.5(7) |  |  |  |  | 5.8(6) |  |  |  |  |  |
| *Pinus echinata* | shortleaf pine |  | 1.2(15) |  |  |  |  |  |  |  |  |  |  |  | 1.1(15) |
| *Pinus elliottii* | slash pine | 3.8(8) | 3.2(8) | 1.2(19) | 1.1(21) |  | 0.8(23) | 2.2(9) | 1.6(17) |  |  | 0.5(30) | 1.7(14) | 5.6(6) |  |
| *Pinus glabra* | spruce pine | 0.9(23) |  | 1.9(13) | 1.3(19) |  |  |  | 1.9(14) |  |  |  |  | 1.8(14) | 0.7(24) |
| *Pinus palustris* | longleaf pine | 1.5(16) | 1(16) | 0.6(25) |  |  | 3.1(9) |  | 1.4(18) |  |  | 3.9(7) | 3.1(8) | 1(23) | 3(7) |
| *Pinus taeda* | loblolly pine | **15.3(1)** | **27.8(1)** | **10.8(3)** | **8.6(3)** |  | **17.7(1)** | **25.1(1)** | 6(7) | **14.9(1)** |  | **14.9(1)** | **19.1(1)** | 4.2(9) | **36.5(1)** |
| *Planera aquatica* | water-elm, planertree | 0.4(39) |  |  |  |  | 0.3(37) |  |  |  |  |  |  |  |  |
| *Platanus occidentalis* | American sycamore | 0.8(26) |  |  | 1(24) |  | 0.4(34) |  |  |  |  | 0.5(32) |  |  |  |
| *Prunus americana* | American plum |  |  |  |  |  |  | 0.6(26) |  |  |  |  | 1(19) |  |  |
| *Prunus serotina* | black cherry | 1.9(15) | 2.5(11) | 2.2(12) | 1.4(18) |  | 2.4(10) | 3.3(7) | 2.7(10) |  |  | 2.9(9) | 3.7(7) | 3.2(11) |  |
| *Prunus spp.* | cherry and plum spp. |  | 1.4(14) |  |  |  |  |  |  |  |  |  |  |  |  |
| *Quercus alba* | white oak | 2.1(13) |  |  |  |  | 1.3(19) |  |  |  |  | 1.5(18) |  |  |  |
| *Quercus falcata* | southern red oak | 1.5(17) | 5.8(6) | 2.6(10) | 0.9(26) |  | 2.1(11) | 1.5(14) | 1.6(16) |  |  | 2.2(13) | 2.8(9) | 1.3(20) | 0.7(25) |
| *Quercus laevis* | turkey oak |  |  |  | 2.1(14) |  |  |  |  | 3.5(10) |  |  | 0.7(24) |  | 1.2(14) |
| *Quercus laurifolia* | laurel oak | 4.9(6) | **8.3(4)** | 3(9) | 5.3(6) |  | 4.1(6) | **7.6(4)** | 3.9(9) | **9.9(4)** |  | 2.1(14) | **10(4)** | 5.2(7) | **5.3(4)** |
| *Quercus lyrata* | overcup oak |  |  | 1.9(14) |  |  | 0.3(39) | 3.1(8) | 0.9(22) |  |  | 0.5(34) | 3.9(6) |  |  |
| *Quercus marilandica* | blackjack oak | 1(21) |  |  |  |  |  |  |  |  |  | 0.4(37) |  |  |  |
| *Quercus michauxii* | swamp chestnut oak |  |  |  |  |  |  | 1(18) |  | 1.7(18) |  |  | 0.8(23) |  | 0.7(26) |
| *Quercus minima* | dwarf live oak | 0.5(33) |  |  |  |  | 0.6(26) |  |  |  |  | 0.5(33) |  |  |  |
| *Quercus nigra* | water oak | **11.3(2)** | **13.4(2)** | **9.4(4)** | **17.4(1)** |  | **11.9(3)** | **12.6(3)** | **15.1(1)** | **13.7(3)** |  | **10.5(3)** | **11.2(3)** | **14.8(1)** | **12.9(2)** |
| *Quercus pagoda* | cherrybark oak |  |  |  |  |  |  |  | 0.7(24) |  |  |  |  |  | 0.7(28) |
| *Quercus phellos* | willow oak | 0.5(32) |  | 0.5(28) |  |  | 0.5(28) |  | 0.6(27) |  |  |  |  | 1.7(15) |  |
| *Quercus sinuata* | Durand oak |  |  |  | 1.3(20) |  |  |  |  |  |  |  |  |  | 1.5(11) |
| *Quercus stellata* | post oak | 0.7(27) | 0.6(20) |  |  |  | 0.5(27) |  |  |  |  | 0.5(31) | 0.7(25) |  |  |
| *Quercus texana* | Texas red oak |  |  |  |  |  |  | 0.8(20) |  |  |  |  | 0.9(22) |  |  |
| *Quercus virginiana* | live oak | 0.4(36) |  |  | 1.5(16) |  | 0.4(31) |  |  |  |  | 0.8(25) |  |  |  |
| *Sassafras albidum* | sassafras |  | 0.6(19) |  |  |  |  | 1.6(13) |  |  |  |  | 1.5(17) |  |  |
| *Taxodium ascendens* | pondcypress | 0.5(30) |  |  |  |  | 0.4(32) |  |  |  |  |  |  |  |  |
| *Taxodium distichum* | baldcypress |  |  |  |  |  | 0.5(29) |  | 0.5(31) |  |  | 0.8(26) |  |  |  |
| *Triadica sebifera* | Chinese tallowtree |  |  | 0.9(21) |  |  | 0.9(21) |  | 1(21) |  |  | 1.7(17) | 0.7(26) |  |  |
| *Ulmus alata* | winged elm | 1.3(19) |  |  |  |  | 1.2(20) | 0.7(22) |  |  |  | 1.3(20) | 0.5(27) | 0.5(29) |  |
| *Ulmus americana* | American elm |  |  |  |  |  |  |  |  | 2.1(14) |  |  |  |  | 0.9(18) |
| *Ulmus rubra* | slippery elm | 0.5(31) |  | 0.6(24) |  |  |  |  |  |  |  |  |  |  |  |
| Importance Value Percent is calculated as the average of relative frequency percent, relative density percent, and relative dominance percent. | | | | | | | | | | | | | | | |
