# Supplementary material for: Long-term effects of catastrophic wind on southern US coastal forests: Lessons from a major hurricane
Source: PLoS One. 2021 Jan 6;16(1):e0243362. doi: 10.1371/journal.pone.0243362 (PMC7787386; doi:10.1371/journal.pone.0243362)
Supplement: S4 Table — Dominance ranking order of the species for each group is presented in parenthesis. Top five dominant species are highlighted. (DOCX) [file pone.0243362.s004.docx]

S4 Table: Importance value percent of seedling (live, dbh<2.54 cm) species for each plot condition group by mid-year of plot inventory period. Dominance ranking order of the species for each group is presented in parenthesis. Top five dominant species are highlighted.

|  |  | Mid-year of plot inventory period | | | | | | | | | | | | | |
| --- | --- | --- | --- | --- | --- | --- | --- | --- | --- | --- | --- | --- | --- | --- | --- |
|  |  | 2002 | | | |  | 2009 | | | |  | 2016 | | | |
| Scientific_name | Common_name | ND | NDBH | ID | IDAH |  | ND | NDBH | ID | IDAH |  | ND | NDBH | ID | IDAH |
| *Acer barbatum* | Florida maple | 0.16(37) | 0.71(25) |  |  |  | 0.48(29) | 0.13(41) |  | 1.17(17) |  | 0.52(23) |  |  | 6.2(6) |
| *Acer negundo* | boxelder |  | 0.71(27) |  |  |  |  | 0.38(31) | 1.27(18) |  |  |  |  |  |  |
| *Acer rubrum* | red maple | **6.71(4)** | **5.23(5)** | **8.9(4)** | 1.96(15) |  | **8.67(4)** | **4.85(5)** | **6.96(5)** | **3.67(5)** |  | **6.96(4)** | 3(7) | 3.91(9) | 2.96(10) |
| *Aesculus flava* | yellow buckeye |  | 0.85(22) |  |  |  |  |  |  |  |  |  |  |  |  |
| *Aesculus glabra* | Ohio buckeye |  |  |  |  |  |  |  | 0.32(33) |  |  |  |  |  |  |
| *Albizia julibrissin* | mimosa, silktree |  |  |  |  |  |  | 0.13(48) |  |  |  | 0.35(34) |  |  |  |
| *Amelanchier spp.* | serviceberry spp. |  |  |  |  |  | 0.16(39) |  |  |  |  |  |  |  |  |
| *Asimina triloba* | pawpaw |  |  |  |  |  |  |  | 0.16(43) |  |  | 0.35(31) | 0.55(25) | 0.18(39) | 0.81(25) |
| *Carpinus caroliniana* | American hornbeam, musclewood | 1.8(15) |  | 1.14(19) | 4.9(8) |  | 5.46(6) |  | 1.58(16) |  |  | 2.96(10) | 0.95(20) | 3.02(12) | 1.62(16) |
| *Carya alba* | mockernut hickory | 0.33(32) | 1.7(17) |  | 1.47(18) |  |  | 1.79(15) | 0.16(37) | 0.44(26) |  | 0.35(30) | 1.77(15) | 0.18(38) | 1.35(18) |
| *Carya aquatica* | water hickory |  |  | 1.83(15) |  |  |  |  |  |  |  | 0.17(42) |  | 0.53(32) |  |
| *Carya carolinae-septentrionalis* | southern shagbark hickory |  |  |  |  |  |  |  |  |  |  |  | 0.14(43) |  |  |
| *Carya cordiformis* | bitternut hickory |  | 1.13(21) |  |  |  |  | 0.38(30) |  |  |  |  | 0.27(33) |  | 0.54(30) |
| *Carya glabra* | pignut hickory | 0.33(31) | 0.28(36) | 0.23(36) | 1.96(16) |  | 0.16(34) | 0.26(35) |  | 0.29(30) |  | 0.52(25) | 0.14(36) | 0.53(30) | 0.81(24) |
| *Carya illinoinensis* | pecan |  |  |  |  |  |  |  | 0.63(25) |  |  |  |  |  |  |
| *Carya ovata* | shagbark hickory |  |  |  | 2.45(14) |  |  |  |  |  |  |  |  |  |  |
| *Castanea pumila* | Allegheny chinkapin | 0.16(38) |  |  |  |  |  |  | 0.16(42) |  |  |  |  |  |  |
| *Catalpa bignonioides* | southern catalpa |  |  |  |  |  |  |  |  | 0.15(41) |  |  |  |  |  |
| *Celtis laevigata* | sugarberry |  |  | 0.46(30) |  |  |  | 0.38(29) |  |  |  | 0.17(39) | 0.14(39) | 0.18(40) | 3.23(9) |
| *Celtis occidentalis* | hackberry |  |  |  |  |  |  | 0.26(37) | 3.16(11) | 0.15(33) |  |  |  | 2.13(15) |  |
| *Cercis canadensis* | eastern redbud |  | 0.42(32) |  |  |  |  | 0.13(47) |  | 1.03(18) |  |  |  |  | 1.08(22) |
| *Chamaecyparis thyoides* | Atlantic white-cedar | 2.13(12) |  | 6.85(6) |  |  | 1.12(18) |  | 5.38(6) |  |  | 0.87(21) |  | 3.02(13) |  |
| *Cornus florida* | flowering dogwood | **4.42(5)** | 3.68(9) | 1.83(13) | 5.88(6) |  | 2.25(13) | 1.4(16) | 0.32(30) | 1.32(15) |  | 0.52(22) | 1.23(18) |  | 1.35(19) |
| *Crataegus crus-galli* | cockspur hawthorn |  |  |  |  |  | 0.48(30) |  |  |  |  |  |  |  |  |
| *Crataegus spp.* | hawthorn spp. | 1.8(16) |  |  | 0.49(28) |  |  |  |  |  |  |  | 0.41(29) | 1.07(23) |  |
| *Diospyros virginiana* | common persimmon | 2.78(10) | 4.95(6) | 2.97(10) | 0.49(23) |  | 4.98(8) | 2.3(13) | 3.96(8) | 0.73(21) |  | 6.09(6) | 2.86(8) | 3.91(10) | 4.04(8) |
| *Fagus grandifolia* | American beech |  | 0.42(33) | 0.46(28) | 2.45(13) |  | 0.16(33) | 0.26(34) | 0.16(39) | 0.59(24) |  |  | 0.14(41) |  | 0.81(27) |
| *Fraxinus americana* | white ash |  |  |  |  |  | 0.64(26) |  |  | 1.32(16) |  | 1.04(19) | 0.95(21) |  |  |
| *Fraxinus pennsylvanica* | green ash | 0.82(23) | 1.41(19) | 0.68(26) |  |  | 0.96(19) | 1.15(18) | 1.27(17) | 0.15(34) |  | 0.52(26) | 0.41(27) | 0.53(29) | 2.7(12) |
| *Ilex opaca* | American holly | 4.26(8) | 4.38(8) | **9.59(2)** | **7.84(5)** |  | 5.14(7) | 2.81(9) | **7.59(4)** | 3.23(6) |  | 5.22(8) | 2.18(10) | **9.59(3)** | 2.7(11) |
| *Juglans nigra* | black walnut |  |  |  |  |  |  |  |  |  |  |  |  | 0.36(36) |  |
| *Juniperus virginiana* | southern redcedar | 0.16(35) |  |  |  |  |  |  | 4.11(7) |  |  |  |  |  |  |
| *Juniperus virginiana* | eastern redcedar | 0.49(28) | 0.14(39) | 0.91(22) | 2.45(12) |  | 2.41(12) | 0.64(23) | 0.63(24) | 1.91(9) |  | 2.26(12) | 1.5(16) | 0.89(24) | 4.58(7) |
| *Liquidambar styraciflua* | sweetgum | **14.24(2)** | **13.58(2)** | 6.39(7) | **14.22(1)** |  | **11.72(2)** | **19.13(1)** | 2.69(12) | **12.48(3)** |  | **11.13(3)** | **12.96(2)** | 4.26(8) | **14.29(1)** |
| *Liriodendron tulipifera* | yellow-poplar | 2.13(13) | 1.27(20) | 0.91(21) | 0.98(19) |  | 0.8(20) | 0.89(21) | 0.47(27) | 0.59(23) |  | 0.35(29) | 0.27(31) | 0.36(33) |  |
| *Magnolia grandiflora* | southern magnolia | 0.82(24) |  |  | 0.49(29) |  | 0.8(21) |  | 0.47(29) |  |  | 1.22(17) |  | 0.71(26) | 0.27(32) |
| *Magnolia macrophylla* | bigleaf magnolia | 0.16(39) |  |  |  |  |  |  | 0.32(32) |  |  |  |  | 0.36(35) |  |
| *Magnolia virginiana* | sweetbay | 4.09(9) | 1.84(13) | **8.9(5)** | 1.47(17) |  | 3.05(11) | 1.02(20) | 3.48(10) | 1.32(14) |  | 1.57(13) |  | 4.62(7) | 1.08(20) |
| *Melia azedarach* | chinaberry |  |  |  |  |  |  | 0.38(32) |  |  |  |  |  |  |  |
| *Morus rubra* | red mulberry |  |  |  |  |  |  |  |  | 0.44(28) |  |  |  |  |  |
| *Nyssa aquatica* | water tupelo |  |  |  |  |  |  |  | 0.16(45) |  |  |  |  |  |  |
| *Nyssa biflora* | swamp tupelo | 1.31(19) | 0.14(37) | 0.23(37) |  |  |  |  | 0.16(41) | 0.15(37) |  |  |  | 0.71(27) |  |
| *Nyssa sylvatica* | blackgum | 1.96(14) | 1.7(15) | 1.6(17) | 0.49(25) |  | 1.12(17) | 1.28(17) | 0.79(22) |  |  | 0.52(27) | 1.77(14) | 1.24(21) | 1.62(17) |
| *Ostrya virginiana* | eastern hophornbeam |  |  |  | 4.9(9) |  | 0.48(27) | 0.13(39) | 0.47(28) | 1.76(11) |  | 0.17(35) | 2.18(11) | 1.24(20) | **8.09(4)** |
| *Oxydendrum arboreum* | sourwood |  | 0.57(29) | 0.46(27) |  |  | 0.16(37) | 0.13(42) |  | 0.15(39) |  | 0.17(36) | 0.27(32) |  |  |
| *Persea borbonia* | redbay | 1.15(21) | 0.71(24) | 3.88(9) |  |  | 4.01(9) | 0.64(22) | **9.65(2)** | 0.88(19) |  | 4.35(9) | 0.68(23) | **6.04(4)** | 0.81(23) |
| *Pinus clausa* | sand pine |  |  |  |  |  |  |  |  | 2.64(7) |  |  |  |  | 0.27(38) |
| *Pinus echinata* | shortleaf pine | 0.16(36) |  |  | 0.49(30) |  |  | 0.13(44) |  | 0.73(22) |  |  |  |  | 0.27(39) |
| *Pinus elliottii* | slash pine | 0.65(26) | 1.98(11) | 1.6(16) | 3.43(10) |  |  | 1.15(19) | 0.47(26) |  |  |  | **4.77(5)** | 1.78(16) |  |
| *Pinus glabra* | spruce pine |  |  | 2.74(12) |  |  | 0.16(36) |  | **7.75(3)** | 0.44(25) |  | 0.52(24) | 0.14(35) | **10.3(2)** | 0.54(28) |
| *Pinus palustris* | longleaf pine | 1.47(17) | 1.84(14) | 1.83(14) |  |  | 0.8(22) | 0.38(27) | 3.96(9) | 1.76(10) |  | 1.04(18) |  | 3.73(11) | 1.89(14) |
| *Pinus taeda* | loblolly pine | **11.62(3)** | **12.02(3)** | **9.36(3)** | **8.33(4)** |  | **8.99(3)** | **15.69(2)** | 2.37(13) | **33.48(1)** |  | **11.13(2)** | **28.24(1)** | **5.15(5)** | **6.47(5)** |
| *Platanus occidentalis* | American sycamore |  |  |  |  |  |  | 0.13(49) |  |  |  |  |  |  |  |
| *Prunus americana* | American plum |  |  |  |  |  | 0.16(40) | 0.38(28) | 0.16(38) | 0.15(36) |  |  | 0.68(24) |  |  |
| *Prunus serotina* | black cherry | 1.47(18) | 1.7(16) | 2.74(11) | 0.49(24) |  | 1.44(16) | 2.42(11) | 1.11(20) | 1.47(13) |  | 1.22(14) | 1.77(13) | 0.53(28) | 1.89(15) |
| *Prunus spp.* | cherry and plum spp. |  | 1.7(18) |  |  |  |  | 0.26(36) |  |  |  | 0.35(33) |  |  |  |
| *Quercus alba* | white oak | 0.49(29) | 0.42(30) |  |  |  | 0.8(25) | 0.64(25) |  | 0.15(38) |  | 0.87(20) | 0.27(30) | 0.89(25) | 0.27(31) |
| *Quercus falcata* | southern red oak | 4.42(6) | 4.81(7) | 1.14(18) | 5.88(7) |  | 3.05(10) | 3.19(8) | 2.37(14) | 2.5(8) |  | 2.78(11) | 3.27(6) | 2.13(14) | 2.16(13) |
| *Quercus incana* | bluejack oak | 0.16(41) | 0.71(26) | 0.23(32) |  |  |  |  |  | 0.15(40) |  | 0.17(37) | 0.14(38) |  | 1.08(21) |
| *Quercus laevis* | turkey oak | 1.31(20) | 0.14(38) |  | 0.98(20) |  | 1.61(15) | 0.64(24) |  | 0.29(29) |  | 1.22(15) | 0.14(34) |  | 0.27(35) |
| *Quercus laurifolia* | laurel oak | 4.42(7) | **6.93(4)** | 5.25(8) | **9.31(3)** |  | **6.42(5)** | **5.61(4)** | 2.06(15) | **5.29(4)** |  | **6.78(5)** | **6.28(4)** | 1.6(18) | **9.43(3)** |
| *Quercus lyrata* | overcup oak |  | 2.83(10) | 0.23(31) |  |  |  | 3.95(6) | 0.16(34) |  |  |  | 2.73(9) | 4.8(6) |  |
| *Quercus margarettiae* | dwarf post oak | 0.16(40) |  |  |  |  |  |  |  |  |  |  |  |  |  |
| *Quercus marilandica* | blackjack oak | 1.15(22) | 0.57(28) | 0.23(33) | 2.94(11) |  | 0.48(28) | 0.13(40) |  |  |  | 0.17(41) | 0.55(26) |  |  |
| *Quercus michauxii* | swamp chestnut oak |  |  |  | 0.49(31) |  |  | 0.13(45) |  |  |  |  | 0.14(42) |  | 0.27(36) |
| *Quercus minima* | dwarf live oak | 2.29(11) | 0.28(34) | 0.23(35) |  |  | 0.8(24) | 0.13(38) |  |  |  | 0.17(40) |  |  | 0.27(37) |
| *Quercus muehlenbergii* | chinkapin oak |  |  |  |  |  |  |  |  |  |  |  |  | 0.36(37) |  |
| *Quercus nigra* | water oak | **15.38(1)** | **14.57(1)** | **11.42(1)** | **9.31(2)** |  | **16.21(1)** | **12.63(3)** | **19.15(1)** | **13.36(2)** |  | **17.39(1)** | **9.96(3)** | **13.5(1)** | **11.59(2)** |
| *Quercus pagoda* | cherrybark oak |  |  |  |  |  | 0.16(41) | 0.13(43) |  |  |  |  |  |  | 0.27(41) |
| *Quercus phellos* | willow oak |  | 0.14(40) | 0.91(23) |  |  | 0.8(23) | 0.26(33) | 1.27(19) | 1.62(12) |  |  | 1.23(19) | 1.78(17) |  |
| *Quercus prinus* | chestnut oak |  |  | 0.23(39) |  |  |  |  |  |  |  |  |  |  |  |
| *Quercus rubra* | northern red oak |  |  |  |  |  |  |  |  |  |  |  |  | 0.18(41) | 0.27(33) |
| *Quercus sinuata* | Durand oak |  |  |  |  |  |  |  |  | 0.29(32) |  |  |  |  | 0.27(40) |
| *Quercus stellata* | post oak | 0.65(27) | 1.98(12) | 0.91(20) |  |  | 0.32(31) | 3.83(7) |  |  |  | 0.35(32) | 1.36(17) |  | 0.27(34) |
| *Quercus texana* | Texas red oak |  |  |  |  |  |  | 1.91(14) |  | 0.44(27) |  |  |  |  |  |
| *Quercus velutina* | black oak |  |  |  | 0.98(22) |  |  |  |  | 0.88(20) |  |  | 0.14(40) |  | 0.81(26) |
| *Quercus virginiana* | live oak | 0.33(33) |  |  | 0.98(21) |  | 0.16(35) | 0.51(26) |  |  |  | 0.17(38) | 0.95(22) |  |  |
| *Sabal palmetto* | cabbage palmetto |  |  |  |  |  |  |  |  |  |  |  |  | 0.18(42) |  |
| *Salix nigra* | black willow |  | 0.85(23) |  |  |  |  |  |  |  |  |  |  |  |  |
| *Sassafras albidum* | sassafras | 0.49(30) | 0.42(31) | 0.23(34) |  |  | 0.16(38) | 2.42(12) | 0.16(36) |  |  | 0.52(28) | 0.14(37) |  |  |
| *Sideroxylon lanuginosum* | chittamwood, gum bumelia |  |  |  |  |  |  |  | 0.16(44) |  |  |  |  |  |  |
| *Taxodium ascendens* | pondcypress |  |  |  |  |  |  |  | 0.79(23) |  |  |  |  | 1.6(19) |  |
| *Taxodium distichum* | baldcypress |  |  | 0.91(24) |  |  |  |  | 0.95(21) |  |  |  |  | 0.36(34) |  |
| *Triadica sebifera* | Chinese tallowtree | 0.33(34) |  | 0.23(38) | 0.49(27) |  |  |  | 0.32(31) |  |  | 5.57(7) |  | 0.53(31) |  |
| *Ulmus alata* | winged elm | 0.82(25) | 0.28(35) | 0.46(29) | 0.49(26) |  | 1.93(14) | 2.42(10) | 0.16(35) | 0.15(35) |  | 1.22(16) | 1.91(12) | 1.07(22) | 0.54(29) |
| *Ulmus americana* | American elm |  |  |  | 0.49(32) |  |  | 0.13(46) |  | 0.29(31) |  |  | 0.41(28) |  |  |
| *Ulmus rubra* | slippery elm |  |  | 0.91(25) |  |  | 0.32(32) |  | 0.16(40) |  |  |  |  |  |  |
| Importance Value Percent is calculated as the relative density percent of the species because of the availability of count data only. | | | | | | | | | | | | | | | |
